# S$\vphantom{0}^{2}$A-RConvNet: standalone self-attention enabled deep learning model for brain tumor classification with MRI images

**DOI:** 10.1038/s41598-026-46010-1

**Published:** 2026-04-25

**Authors:** Uttam Waghmode, Ashwini Naik, Jyoti Deone, Somdotta Roy Choudhury, Dhanashri Dhawale, Digambar Puri, Surendra Solanki

**Affiliations:** 1 Ramrao Adik Institute of Technology, Navi Mumbai, India; 2 Lokmanya Tilak College of Engineering, Koparkhairane, Navi Mumbai, India; 3 Department of Artificial Intelligence and Machine Learning, Manipal University Jaipur, Jaipur, 303007 Rajasthan India; 4 Department of Information Technology, Vidhyalankar Institute of Technology, Wadala, Mumbai, India

**Keywords:** Cancer, Computational biology and bioinformatics, Health care, Mathematics and computing

## Abstract

Globally, the main factor that contributes to increasing the mortality rate among people is the development of abnormal cells in the brain, which leads to a Brain Tumor (BT). Therefore, the classification of BT is essential to prevent the increasing death rate by diagnosing the tumor based on its type. In order to classify the types of BT, several models are introduced, but they possess numerous drawbacks, including poor accuracy, higher time consumption, computational complexities, overfitting, and so forth. Hence, the Standalone Self-Attention based Repeated Convolutional Network ($\hbox {S}^{2}$A-RConvNet) model is developed to classify the BT types accurately to save the lives of affected people by solving the limitations of conventional approaches. The incorporation of the Standalone Self-Attention ($\hbox {S}^{2}$A) module enables the RConvNet to focus more on the tumor area, which helps to increase the model’s accuracy in BT categorization. Furthermore, the extraction of Structured ResNet Attention Gray-level (SRAG) features increases the training period and decreases the computational complexities, which leads to better performance of the model in BT classification. The $\hbox {S}^{2}$A-RConvNet model attained the values of sensitivity of 97.61%, precision of 98.71%, F1-Score of 98.16%, specificity of 98.43% and accuracy of 97.98% with 90% of training using the BraTS 2021 dataset.

## Introduction

The human brain is considered one of the most important organs as it regulates several processes like vision, memory, breathing, movement, response, and emotions^1^. These processes would be interrupted if any tumor originates in the brain^2^. BT is considered a key factor in mortality rates worldwide and is most common in both children and adults^3^. The symptoms of a BT include vomiting, headaches, speaking and walking troubles, mental health complications, eye issues, and others^4^. Most BTs arise in the brain, while others evolve from other parts of the body. The WHO categorizes BT into 4 grades based on their intensity of aggression, such as grade-1, 2, 3 and 4 tumors^5^. Glioma is a general instance of this higher-grade tumor^6^. BT affects people regardless of their age and gender. Risk factors of BT include specific hereditary illnesses, ionizing radiation exposure, and genetic disposition^7^. Nevertheless, the exact reason for cause of BT remains unclear^8^. Developments in diagnosis, radiation treatments, and surgeries have increased the survival rates. Regardless of these advancements, they remain major health concerns^9^.

The survival rate of individuals with a BT relies heavily on timely diagnosis and efficiency of the prognostic treatment^10^. Traditional diagnostic methods like biopsy are invasive, and their ability is limited to detecting early-stage tumors^11^. Non-invasive imaging tests, including positron emission tomography (PET), CT scans, and magnetic resonance imaging (MRI), are employed to diagnose and evaluate the internal conditions^12^. Amidst, MRI acquires high contrast details about the tumors and is regarded as the preferred option^13^. Compared to CT scans, MRI provides numerous benefits, including the capacity to track every region of the brain while scanning, and improved image contrast^14^. Traditionally, MRI is reliant on a medical expert’s observation^15^. Hence, it is prone to several drawbacks like human errors, more time consumption, inconsistencies due to noisy data, and labour intensity. This entails the adoption of automated computer-aided diagnostic (CAD) methods^16^.

Considering the diverse morphologies and complex analytical structures, it is difficult to detect BT with high accuracy. Adding to that, the noise and falsification made by the acquisition obstruct the MRI equipment and make the segmentation and Region of Interest (ROI) process inconvenient^17^. Artificial Intelligence (AI) and its subgroups, including feature extraction, selection, and reduction, have become crucial in identifying BT among neurosurgeons^18^. Traditional Machine Learning (ML) techniques depend on manual features, which disrupts the robustness and durability of the solution^19^. These approaches exhibit several drawbacks, including bulky models, inadequate precision, high computational cost, and privacy concerns^20^. Deep Learning (DL) methods encompassing complex architectures offer powerful capacities for distinct tasks. However, they are difficult to interpret and require high computational cost^21^. The Convolutional Neural Networks (CNN) have proven efficient in processes like tumor detection, segmentation, and classification, helping physicians in complex tasks^22^. For instance, the You Only Look Once (YOLO) series, which utilizes CNN, possesses high processing speed and accuracy in image processing tasks^23^. Nevertheless, they still face shortcomings in reducing false positives and differentiating classes, which is pivotal in clinical practices^24^.

Therefore, the S$\vphantom{0}^{2}$A-RConvNet model is developed to categorize the types of BT by eliminating the shortcomings of traditional methods. The utilization of the Standalone Self-Attention based U-Net (S2A-U-Net) model for the segmentation process increases the accuracy of the S2A-RConvNet because of its capability to precisely segment the tumor region. Moreover, the computational load is minimized by cropping the tumor region through the incorporation of the process of ROI extraction. The SRAG feature extraction diminishes the computational complexity and processing time by extracting suitable features for accurate BT classification. The main contribution of the proposed model is provided below.

**Standalone Self-Attention-based Repeated Convolutional Network** (S$\vphantom{0}^{2}$**A-RConvNet) Model:** The S2A module is combined for the process of segmentation, feature extraction, and classification, which boosts the model’s capability to comprehend the complex boundaries and shapes of the tumor region. Moreover, the assimilation of the S$\vphantom{0}^{2}$A module with RConvNet makes the model more capable of capturing long-range relationships, which leads to improved BT classification accuracy. Specifically, the advantage of these combined techniques enhances the model’s performance while reducing computational complexity compared with other existing models.

The research article is structured as follows: Section 2 provides a review of existing works and challenges faced by the existing approaches, Section 3 covers the detailed description of the proposed model, experimental results are presented in Section 4, and lastly, Section 4 concludes the research article along with future work.

## Literature survey

The methodologies, benefits, and drawbacks of the related articles for BT detection and classification are illustrated below,

Mohamed Wageh et al.^1^ developed a robust approach to detect BT, which integrates pre-trained CNN models, transfer learning with multiple pre-trained models to get deep features and the utilization of a generic algorithm for feature selection reduced the computational cost. However, the suggested approach needs a long time period to process the huge amount of data. Anantharajan, S. et al.^2^ introduced an Ensemble Deep Neural Support Vector Machine (EDN-SVM) model to detect the BT in its early stage. Here, the utilization of Fuzzy c-means-based segmentation and Gray-level co-occurrence matrix (GLCM) extracts salient and significant features, which consume less time. The major drawback in this approach is the usage of grayscale photographs, which affects the segmentation process.

Muksimova, S. et al^3^ developed the YOLOv5m model to detect the BT, where the Enhanced Spatial Attention layer (ESA) was integrated to minimize the rate of false positives and increase the precision for identifying the various kinds of tumors. However, the YOLOv5m model does not apply to other imaging modalities other than MRI. Agarwal, M. et al^4^ introduced a Modified Inception V3 model to detect the BT correctly. The contrast enhancement approaches were integrated with the deep transfer learning methods, which increased the accuracy in detection. The suggested model failed to solve the problems, such as bias and the absence of robustness.

Mahesh, T.R. et al^5^ suggested the EfficientNetB0 to provide precise results in BT detection. The employment of an explainable technique provides visualization of valuable insights into decision-making that enriches understanding and trust. However, the EfficientNetB0 model suffered from handling complex images, which limits its performance. Aamir, M. et al^6^ developed an optimized CNN model to identify the BT accurately. The stochastic gradient descent (SGD) and Adam were employed to augment the hyperparameters of the identifier. The model’s complexity was minimized by the fine-tuning of the hyperparameters. But the model struggles with the overfitting issues.

Preetha, R. et al^7^ introduced the Deep Convolutional Neural Network (DCNN) with customized layers to detect the BT. The performance was increased by tuning with a Bayesian optimization algorithm. However, the model cannot extract information-rich feature maps and fails to provide better results. Guluwadi, S. et al.^8^ introduced the ResNet-50 model for BT detection, where the Grad-AM model was fused with the model to provide an explainable and transparent framework. The Elastic deformation technique was utilized to augment the images, and resolves the black box issue using Grad-CAM. The size and diversity of the dataset were limited, which impacts the model’s generalization ability.

Hafiz Muhammad.TK et al.^13^ presented a customized pretrained EfficientNetB7 model for BT classification. Here, the effective preprocessing is performed by FastNIMeans Denoising Colored filter and to improve the quality of the input. The involvement of multiple pretrained models provides significant feature extraction that boosts the accurate classification. However, the model has high computation time due to a lack of segmentation and optimization. Hussein Alshaari and Saeed Alqahtani [14] developed a Deep-EFNet model by combining the EfficientNetB0 with transfer learning for BT classification. The application of dropout and L2 regularization mechanisms moderates the overfitting issues. Although the DL model overcomes the class imbalance issues and provides better adaptability, still affected by misclassification. The summarization of recent literatures is given in Table 1.

**Table 1:** Literature review summary of recent research used for BT classification.

Author’s Name	Method	Strength	Limitations	Achievements
Mohamed. W et al.^1^	Pretrained CNN models	Extracting the important features in a wide range improves the model’s performance.	Handling large data requires high computation time.	Accuracy of 99.7%
Anantharajan. S et al.^2^	EDN-SVM	High generalizability with less computation time.	The combination of ML and DL models creates complexity and causes false errors.	Accuracy of 97.93%, sensitivity of 92 %, and specificity of 98 %.
Muksimova. S et al^3^	Yolov5m model	The augmentation techniques enhanced performance and generalization from diverse data.	Attained a lower confidence level and computational overheads, which led to poor reliability on unknown BT.	Precision of 92%, Recall of 88% and mAP50 of 92%.
Agarwal. M et al^4^	Auto Contrast Enhancer	Provide a robust structure for automated BT classification	Poor adaptability and a required large response time.	Accuracy of 98.89%, sensitivity of 95.28% and specificity of 94.47%.
Mahesh. TR et al^5^	XAI-enhanced efficientNetB0 model	Provide a reliable diagnosis with better decision-making ability.	Struggled with real-world clinical adoption.	Accuracy of 98.72%, precision and recall above 97%.
Aamir. M et al^6^	Optimized CNN	Generate reliable and transformative outcomes for medical diagnostics	Overfitting is caused by the model’s complexity, which limits the generalizability.	accuracy of 97.18%, average precision, recall, and f1-score values of 97%.
Preetha. R et al^7^	EfficientNet-B4 CNN model	Lightweight architecture facilitates better robustness and generalization.	The model struggled with high computational complexity and error rate.	accuracy of 99.33%, Precision of 98.68%, Recall of 100% and specificity of 98.67%.
Guluwadi. S et al^8^	explainable AI using Grad-CAM with Resnet 50	Augmentation techniques mitigating overfitting risks. Grad-CAM ensures transparency in decision-making.	A constrained and diverse dataset limits the generalization and interpretability.	accuracy of 98.52% and precision-recall metrics exceeding 98%
Hafiz Muhammad.TK et al.^13^	Pretrained EfficientNetB7 Model	The data augmentation technique reduced the overfitting problem and ensured fast learning. Training.	Lack of segmentation required a high processing time.	Accuracy of 98.97%, precision of 93.76%, recall of 93.53%, F1-score of 93.26%, MIOU of 95.73% and a lower miss classification rate of 1.02%.
Hussein Alshaari and Saeed Alqahtani^14^	Deep-EFNet	Transfer learning and dual regularization provides robust performance and stabilizes the training.	The model has misclassification, which affects the accurate classification.	Accuracy of 98%, macro average for Precision 0.96, Recall 0.95, and F1-score 0.95.

###  Challenges

- The transfer learning integrating multiple pre-trained deep CNN models^1^ has proved efficient in assisting professionals in the diagnosis of BT. However, it lacked diverse datasets for model training.
- The EDN-SVM classifier^2^ extracted essential features in a shorter period compared to conventional approaches. However, it used only grayscale photographs, which affects the segmentation process.
- The EfficientNetB0 architecture with Grad-CAM^5^ offered valuable insights into decision-making. However, it suffered from handling complex images.
- The ResNet50 model^8^ resolved the black box issue by providing insights and visualization. The limitation of this approach is the lack of diverse datasets.

### Problem statement

The brain is one of the complex organs of our human body, so it is very complex to diagnose BT. The process of tumor segmentation is a challenging task in BT classification, and many studies have been done to slice the tumor region, but due to the tumor shape, texture, border, and irregularity, they failed to accomplish the segmentation accurately and required high time consumption. The noisy factors and unrelated information in the extracted features affected the classification accuracy. Therefore, the S$\vphantom{0}^{2}$2A-RConvNet is introduced to categorize the BT in this research by solving the existing limitations in BT classification. The BT classification process begins with the collection of brain MRI images, which are taken from the BraTS 2021^25^ and BraTS 2017 dataset^26^, respectively. Afterward, the segmentation process is accomplished to produce the segmented tumor mask, and then the image regions are identified and extracted using ROI extraction. Subsequently, the features are extracted from the segmented tumor mask and fed into the DL model to provide classification results.

## Brain tumor classification using standalone self-attention-based repeated convolutional network

**Fig. 1:**
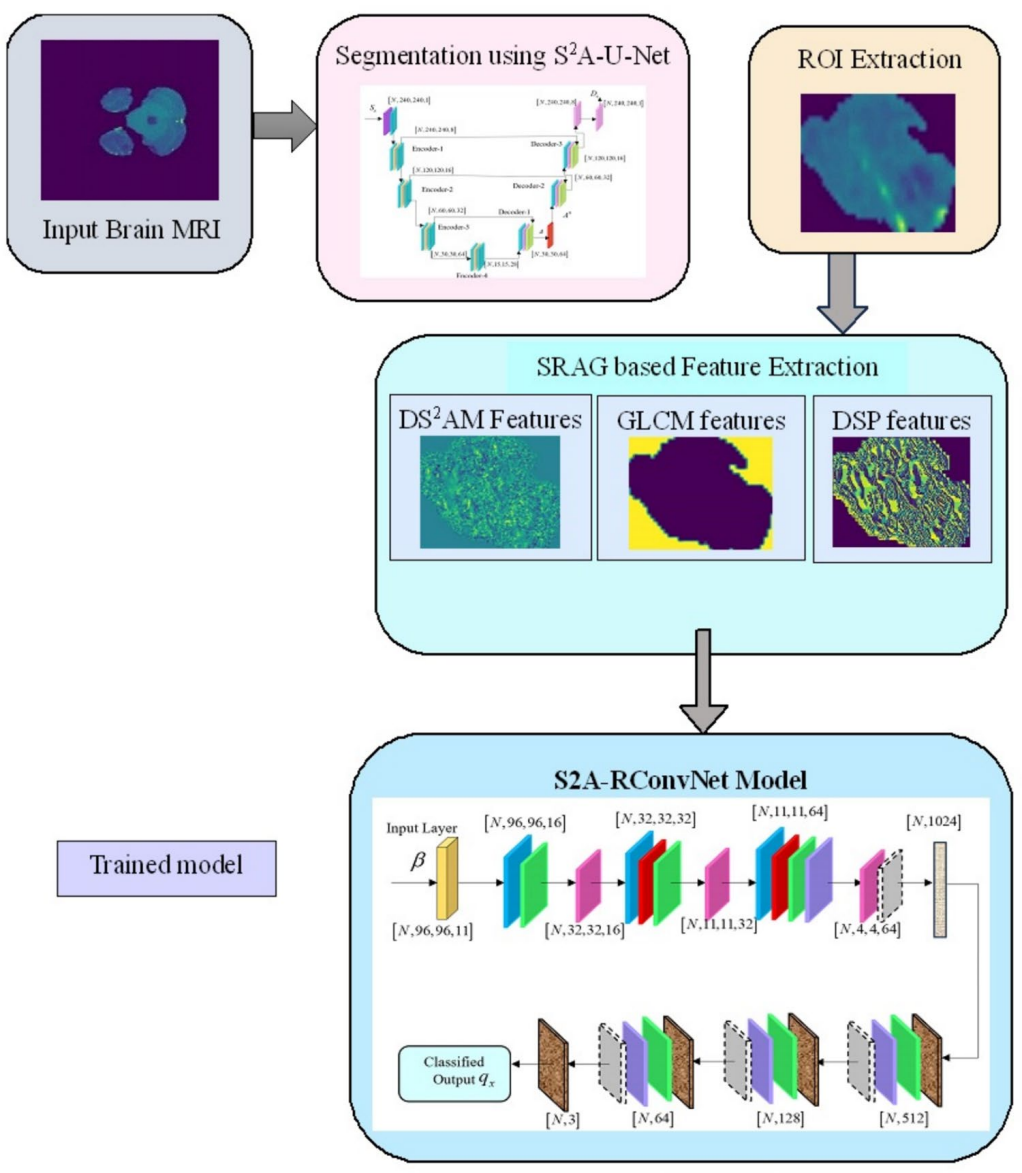
Overall working of the S$\vphantom{0}^{2}$A-RConvNet model for Brain Tumor Classification.

The classification process of the BT using the S$\vphantom{0}^{2}$2A-RConvNet model is initialized by collecting the brain MRI images from the datasets, including the BraTS 2021 and the BraTS 2017 datasets, respectively. Afterward, the tumor areas are segmented by the S2A-U-Net model, where the S$\vphantom{0}^{2}$ 2 A module is incorporated with the model to enhance the segmentation process. After segmenting the correct tumor regions, the ROI extraction process is accomplished by cropping the tumor region from the image, which aids in decreasing the computations by removing the unwanted regions. Subsequently, the SRAG features are extracted, and it is the combination of several features, such as Deep Standalone Self-Attention based Matrix (DS2AM) features, GLCM features, and Deep Structural Pattern (DSP) features, respectively. Afterward, the S$\vphantom{0}^{2}$2A-RConvNet model classifies the BT by taking SRAG features as input, which is also the combination of the S$\vphantom{0}^{2}$ 2 A module and the Repeated CNN model. Finally, the S$\vphantom{0}^{2}$2A-RConvNet model classifies the BT into 3 different classes, including Necrotic Core (NCR), Peritumoral Edema (ED), and Enhancing Tumor (ET), respectively. The working of the S$\vphantom{0}^{2}$2A-RConvNet is exposed in Fig. 1.

### Input image acquisition

The BT classification process using the S$\vphantom{0}^{2}$2A-RConvNet model starts by gathering brain MRI images from the two datasets, with the size of which is mathematically symbolized as,

1 $$\begin{aligned} Q=\{S_{1},S_{2},...S_{e},...S_{L}\} \end{aligned}$$

where $S_{L}$ is the number of image counts in *Q* dataset and the $e^{th}$ brain MRI image is denoted as $S_{e}$ with dimension [*N*, 240, 240, 1], which is forwarded to the segmentation model. The input brain MRI images are mathematically notated as,

2 $$\begin{aligned} Q=\{S_{e}\}^{L}_{e=1} \end{aligned}$$

where $S_{e}$ represents the $e^{th}$ brain MRI image of the dataset *Q* and *L* indicates the total number of brain MRIs.

### Segmentation using standalone self-attention-based U-Net model

**Fig. 2:**
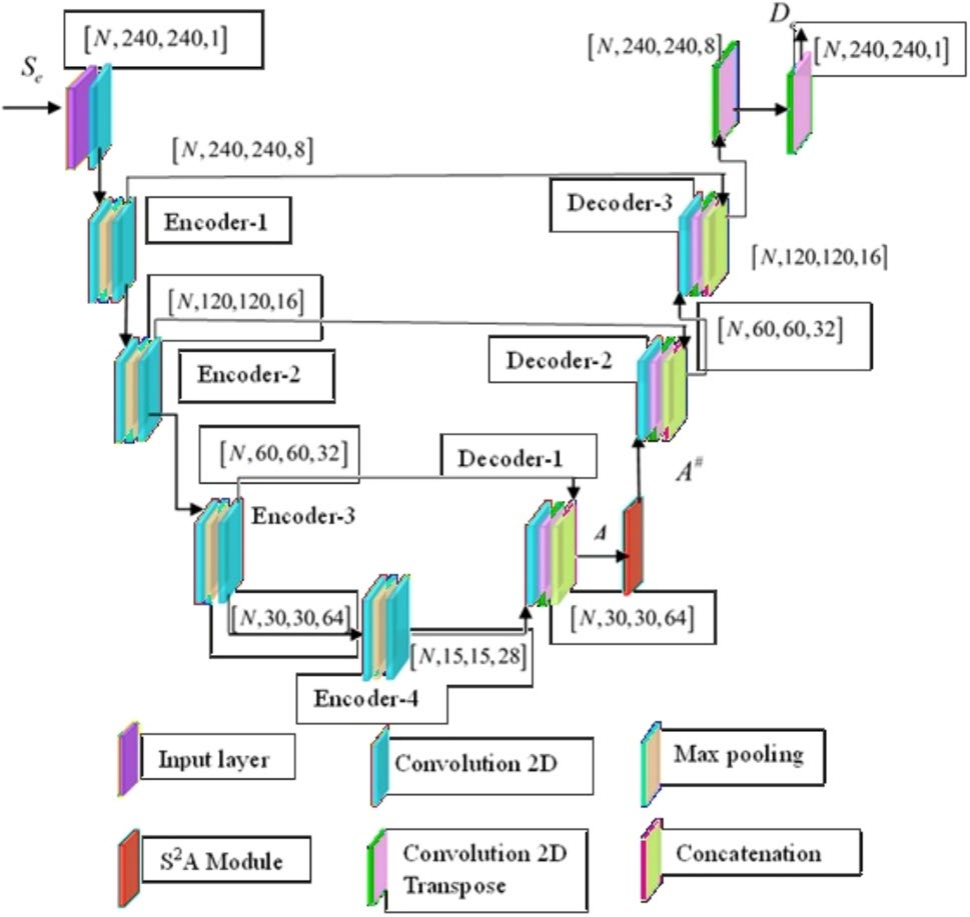
S$\vphantom{0}^{2}$A-U-Net Model’s Architecture.

The segmentation is the method of segregating the brain MRI image into a group of pixels with the same features, which provides crucial information for BT classification. The single brain MRI image comprises several slices, so the manual segmentation takes more time to process, and it is a challenging task. Hence, the S$\vphantom{0}^{2}$2A-U-Net is used for segmentation, which solves the difficulties related to the manual segmentation process. The S$\vphantom{0}^{2}$2A-U-Net is the combination of the U-Net and the S$\vphantom{0}^{2}$ 2 A module, where the assimilation of the S2A module empowers the U-Net to focus only on the relevant region, and the S$\vphantom{0}^{2}$2A-U-Net model can extract global and local features at different sizes, leading to improved results in the segmentation. The S$\vphantom{0}^{2}$2A-U-Net model’s architecture is revealed in Fig. 2

Initially, the input brain image $S_{e}$ is given to the S$\vphantom{0}^{2}$A-U-Net, which is a two-stage model that contains a decoder and encoder units. The S$\vphantom{0}^{2}$A-U-Net model consists of pooling layers, transposed convolution layers, concatenation layers, and convolution layers. The feature maps from the contracting path are transferred to the expanding path using a concatenation operation. The convolutional and pooling layer of the encoder is used to downscale the input’s size, and every down-sampling step diminishes the feature map’s spatial size. The output of the convolutional layer $\partial$ is mathematically represented as,

3 $$\begin{aligned} \partial =f(\phi *S_{e}+\rho ) \end{aligned}$$

Here $\partial$ is the resultant map from the first convolutional layer, *f* represents the size of the kernel, $*$ signifies the convolutional operation, $\phi$ and $\rho$ are the weight and bias parameters, respectively. The transposed convolution layer of the decoder is used to upscale the size of the features, and this process helps to retain the feature map’s spatial resolution. The working of the transposed layer $\alpha$ is notated as,

4 $$\begin{aligned} \alpha = f(\phi ^{\#}*S_{e}+\rho ) \end{aligned}$$

Where $\alpha$ is the feature map of the first transposed layer, $\phi ^{\#}$ indicates the layer’s transposed weight parameter. The generated feature map $\alpha$ is fused with the convolutional layer of the encoder in the concatenation layer, and its output *A* is presented to the S$\vphantom{0}^{2}$A module. The detailed working of and its working of S2A module is provided in section 3.6. Subsequently, the S2A module’s output $A^{\#}$ is fed into another set of transposed convolutional layers of the decoder. Finally, the segmented tumor region $D_{e}$ with size of [*N*, 240, 240, 1] is obtained from the final convolutional layer of the S$\vphantom{0}^{2}$A-U-Net model, which is then passed to the phase of ROI extraction.

### Region of interest extraction

The ROI extraction is a significant step in BT classification to minimize the processing time by cropping the tumor area and removing the irrelevant areas from the segmented tumor region $D_{e}$. Moreover, this extraction facilitates lessening the computational load by applying denoising and removing artifacts, which leads to better quality images. After extracting the ROI from the segmented tumor region, the resultant image $Z_{e}$ of size [*N*, 240, 240, 1] is forwarded into the feature extraction process.

### Structured ResNet attention gray-level based feature extraction

Each ROI extracted image $Z_{e}$ has its own significant features, which play a supportive role in accurate BT classification. Thus, the SRAG features are extracted from $Z_{e}$, which is the integration of multiple features, including DS$\vphantom{0}^{2}$AM features, GLCM features, and DSP features. The accuracy of the S$\vphantom{0}^{2}$A-RConvNet model in classifying BT is increased by successfully extracting the SRAG features. Moreover, the extraction of SRAG features offers the advantages of reducing overfitting, computational complexity, and accelerating the training process.

#### Extraction of deep standalone self-attention-based matrix features

The DS$\vphantom{0}^{2}$AM features are extracted through combining the pre-trained ResNet-101 model with the S$\vphantom{0}^{2}$A module. The employment of a pre-trained ResNet-101 model to extract features saves significant time because it extracts the features based on the existing learned knowledge. The ResNet-101 is a CNN-based model, which is the improved version of the ResNet-50 model, and it contains a total of 101 layers. The number of filters in the layers of ResNet is the same as the dimension of the output feature map. The significant feature of the Resnet-101 model is that it improves the residues among the properties of desired convolution and input, so the desired features are attained effortlessly compared to other models. The ROI extracted image, $Z_{e}$ given to the first layer of ResNet-101, and here, the feature map, *b* is taken from layer 3 of the ResNet-101 model and handed into the S$\vphantom{0}^{2}$A module, and the output $b^{\#}$ size [*N*, 112, 112, 64] is obtained from the S2A module, where its working is discussed in section 3.6. Afterward, the GLCM features, such as correlation, maximum probability, cluster shade, Angular Second Moment (ASM), and cluster prominence, are extracted from the feature $b^{\#}$, which are clearly described as follows.

**Correlation:** It helps to explicate and determine the spatial dependencies among the pixels of the feature $b^{\#}$ and it is denoted as,

5 $$\begin{aligned} W_{1}=\sum ^{M}_{y=1} \sum ^{M}_{z=1} \frac{(y-E_{y})(z-E_{z})}{\sqrt{F_{y}\times F_{z}}} \end{aligned}$$

where, *y* and *z* indicates the pixels, $W_{1}$ denotes the correlation feature of size [*N*, 96, 96, 1], *M* is the number of gray levels, $F_{y}$ and $F_{z}$ represents the standard deviation in the horizontal and vertical spatial domain, similarly, $E_{y}$ and $E_{z}$ signifies the mean in the horizontal and vertical spatial domain.

**ASM:** It estimates the recurrences in the pixel pairs, and the highest value of ASM is obtained when the distribution of the gray level is constant, which is denoted as,

6 $$\begin{aligned} W_{2}=\sum ^{M-1}_{y=1} \sum ^{M-1}_{z=1}U(y,z)^{2} \end{aligned}$$

where *U*(*y*, *z*) denotes the normalized value, and $W_{2}$ represents the ASM feature of size [*N*, 96, 96, 1].

**Maximum Probability:** It estimates the maximum possibility of creating the interest pixels, which is denoted as,

7 $$\begin{aligned} W_{3}=Max [U(y,z)] \end{aligned}$$

where $W_{3}$ indicates the maximum probability feature of size [*N*, 96, 96, 1

**Cluster Shade:** It estimates the matrix’s skewness and measures the perceptual impression of uniformity, and it is represented as,

8 $$\begin{aligned} W_{4}=\sum ^{M-1}_{y=1} \sum ^{M-1}_{z=1}(y+z-E_{y}-E_{z})^{3}U(y,z) \end{aligned}$$

Where $W_{4}$ signifies the cluster shade feature of size [*N*, 96, 96, 1]

**Cluster Prominence:** It is also an estimation of asymmetry, and the matrix around the value of the mean is a peak when the value of cluster prominence is, and it is represented as.

9 $$\begin{aligned} W_{5}=\sum ^{M-1}_{y=1} \sum ^{M-1}_{z=1}(y+z-E_{y}-E_{z})^{3}U(y,z) \end{aligned}$$

where $W_{5}$ indicates the cluster prominence feature of size [*N*, 96, 96, 1]. Lastly, the DS$\vphantom{0}^{2}$AM features are attained by concatenating GLCM features, and it is denoted as,

10 $$\begin{aligned} W=\{W_{1}||W_{2}||W_{3}||W_{4}||W_{5}\} \end{aligned}$$

where *W* signifies the DS$\vphantom{0}^{2}$2AM features with dimension [*N*, 96, 96, 5].

#### Extraction of deep structural pattern features

The DSP features are extracted $Z_{e}$ using the pretrained ResNet-152, and the Grid-based Structural Pattern (GSP) is applied on the ResNet-152 model’s output from layer 3. The ResNet-152 is a 152-layer deep CNN model, which contains a sequence of batch Normalization (BN), convolutional, and activation layers with a sequence of residual blocks, and every residual block comprises 2 convolutional layers with BN and activation. Moreover, the ResNet-152 model functions according to the generalization of residual learning, which enables the network to acquire residual functions. The feature map *k* is taken from layer 3 of the ResNet-152 model, and it has a size of [*N*, 112, 112, 64]. Afterward, the GSP is applied to the feature map *k*, which is the upgraded version of Local Binary Pattern (LBP) descriptors. The drawback of intensity difference in the LBP is overcome using the GSP by measuring the value of gray level by assuming each pixel as the center pixel, thus 9 gray level values are obtained, and lastly mean value is estimated for all the values of gray level to gain the DSP feature, *O* of size [*N*, 96, 96, 1] respectively.

#### Extraction of gray level co-occurrence matrix features

The GLCM is one of the most popular and simplest processes to extract the textural features by determining the textural relationship among the pixels through executing the operation based on the second-order statistics. The GLCM features, including entropy, dissimilarity, energy, homogeneity, and contrast, are extracted and concatenated to form the feature *B* with a size of [*N*, 96, 96, 5]. The description of GLCM features is shown in Table 2.

**Table 2:** Comparison of Time taken for rotation, Zoom in and Zoom out.

Feature	Description	Expression	Size
Energy	It is the quantifiable sum of the recurrences for a pair of pixels.	$B_{1}=\sqrt{\sum ^{M-1}_{a=1}\sum ^{M-1}_{b=1} T(a,b)^{2}}$ where $B_{1}$ indicates energy, and *T*(*a*, *b*) is the Gray-scale’s normalized value at the pixel *a* and *b* of the image $Z_{e}$	[*N*, 96, 96, 1]
Homogeneity	It computes the local consistency of the image.	$B_{2}=\sum ^{M-1}_{a=1} \sum ^{M-1}_{b=1} \frac{T(a,b)}{1+(a-b)^{2}}$ where $B_{2}$ represents the homogeneity	[*N*, 96, 96, 1]
Dissimilarity	It measures the gray level mean variance in the image’s distribution.	$B_{3}=\sum ^{M-1}_{a=1} \sum ^{M-1}_{b=1}|a-b|T(a-b)$ where $B_{3}$ signifies the dissimilarity	[*N*, 96, 96, 1]
Contrast	It calculates the image’s spatial frequency and determines the image’s local variations.	$B_{4}=\sum ^{M-1}_{a=1} \sum ^{M-1}_{b=1} |a-b|^{2}T(a-b)$ Where $B_{4}$ denotes the contrast	[*N*, 96, 96, 1]
Entropy	It is used to measure the randomness of the image.	$B_{5}=\sum ^{M-1}_{a=1} \sum ^{M-1}_{b=1} T(a-b)log_{2}T(a,b)$ Where $B_{4}$ is the entropy.	[*N*, 96, 96, 1]
	**GLCM Features**	$\{B_{1}||B_{2}||B_{3}||B_{4}||B_{5}\}$ where *B* indicates the extracted GLCM features	[*N*, 96, 96, 5]

At last, the extracted features of DS$\vphantom{0}^{2}$AM are represented as *W*, GLCM is represented as *O*, and DSP is represented as *B*, which are concatenated to attain the significant SRAG features is expressed as,

11 $$\begin{aligned} \beta =\{W||O||B\} \end{aligned}$$

Here, $\beta$ denotes the SRAG features of size [*N*, 96, 96, 11], which are handed to the S$\vphantom{0}^{2}$A-RConvNet model to classify the BT correctly.

### Standalone self-attention-based repeated convolutional network for brain tumor classification

**Fig. 3:**
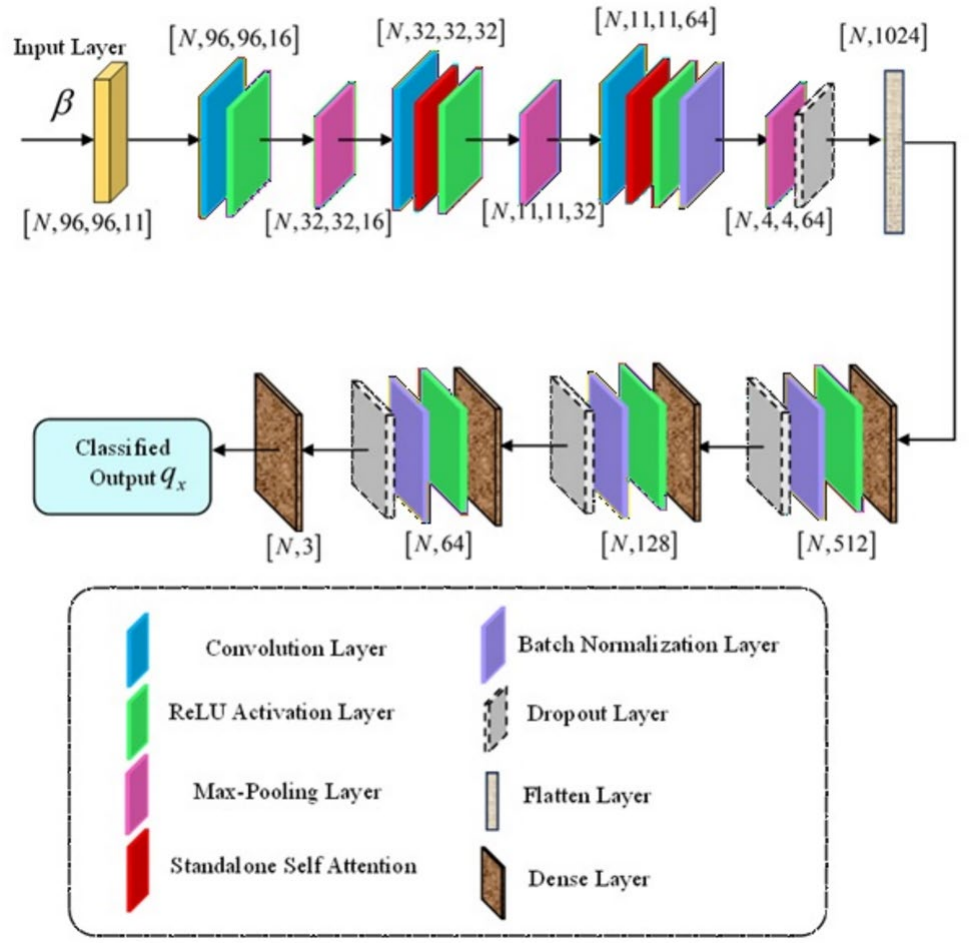
Architecture of the S$\vphantom{0}^{2}$A-RConvNet model.

The S$\vphantom{0}^{2}$A-RConvNet is developed to categorize the BT using brain MRI images, which incorporates a repeated CNN model and the S$\vphantom{0}^{2}$A module. It contains a series of layers, such as a convolution, pooling, dense, activation, a BN, and a dropout layer. Primarily, the SRAG extracted features $\beta$ with the size of [*N*, 96, 96, 11] are fed to the RConvNet model’s input layer, and subsequently, it is passed to the layer of convolution, which is the vital unit of the RConvNet model. The filters of the convolutional layer with various shapes and sizes are utilized to extract diverse local features through the backward and forward propagation from the feature $\beta$. The S$\vphantom{0}^{2}$A-RConvNet model’s architecture is depicted in Figure 3.

Moreover, the convolutional layer works by applying a sequence of convolutional filters to generate feature maps by learning features, which is represented as

12 $$\begin{aligned} P= d \sum (\beta *G) \end{aligned}$$

where *P* denotes the convolution layer’s feature map, $*$ is the operation of convolution, *d* implies the bias function, and *G* signifies the convolution filters. The feature map *P* is passed to the ReLU activation layer and it is selected due to its simplicity and capability of solving the vanishing gradient issue throughout the training process. The ReLU activation function is mathematically defined as,

13 $$\begin{aligned} ReLU(P)=max(0,P) \end{aligned}$$

where *max*(0, *P*) implies the maximum function. Followed by the activation function, there is a pooling layer, which enables the training process of the RConvNet to be quicker and reduces the network’s memory size by minimizing the connections over the layer of convolutions, and here, max pooling operation is used to decrease the feature map’s dimensionality. Subsequently, the pooling layer’s output is provided to the next convolutional layer, and further, its resultant $P^{\#}$ of size [*N*, 32, 32, 32] is fed into the S$\vphantom{0}^{2}$A module, where its working is provided in section 3.6. Later, the output feature map of the S$\vphantom{0}^{2}$A module *H* is then fed into a series of activation, pooling, and convolutional layers. The next resultant feature map of the convolutional layer, *J* of size, [*N*, 32, 32, 32] is again passed into the S$\vphantom{0}^{2}$A module, and its output $J^{\#}$ is transferred to the activation layer and then the BN layer, which is used to attain enhanced outcomes and also helps to accelerate the convergence. The dropout layer is present, followed by the BN layer, which facilitates avoiding overfitting in the RConvNet model and improves the normalization ability of the model. This layer’s output is passed into the flatten layer, and it helps to minimize the feature map size into a single dimension of [*N*, 1024].

Finally, the last dense layer of the RConvNet model provides the BT classification output, $q_{x}$ of size [*N*, 3], which categorizes the BT into 3 different classes, namely, NCR, ET, and ED, respectively. The error between the model’s prediction and the actual values is quantified loss function, which is represented as,

14 $$\begin{aligned} X_{loss}=- \sum ^{Z}_{x=1}g_{x}log(q_{x}) \end{aligned}$$

where $x_{loss}$ signifies the Categorical Cross Entropy, *Z* represents the total classes, $g_{x}$ indicates the true label, and $q_{x}$ predicted value, respectively.

### Standalone self-attention module

**Fig. 4:**
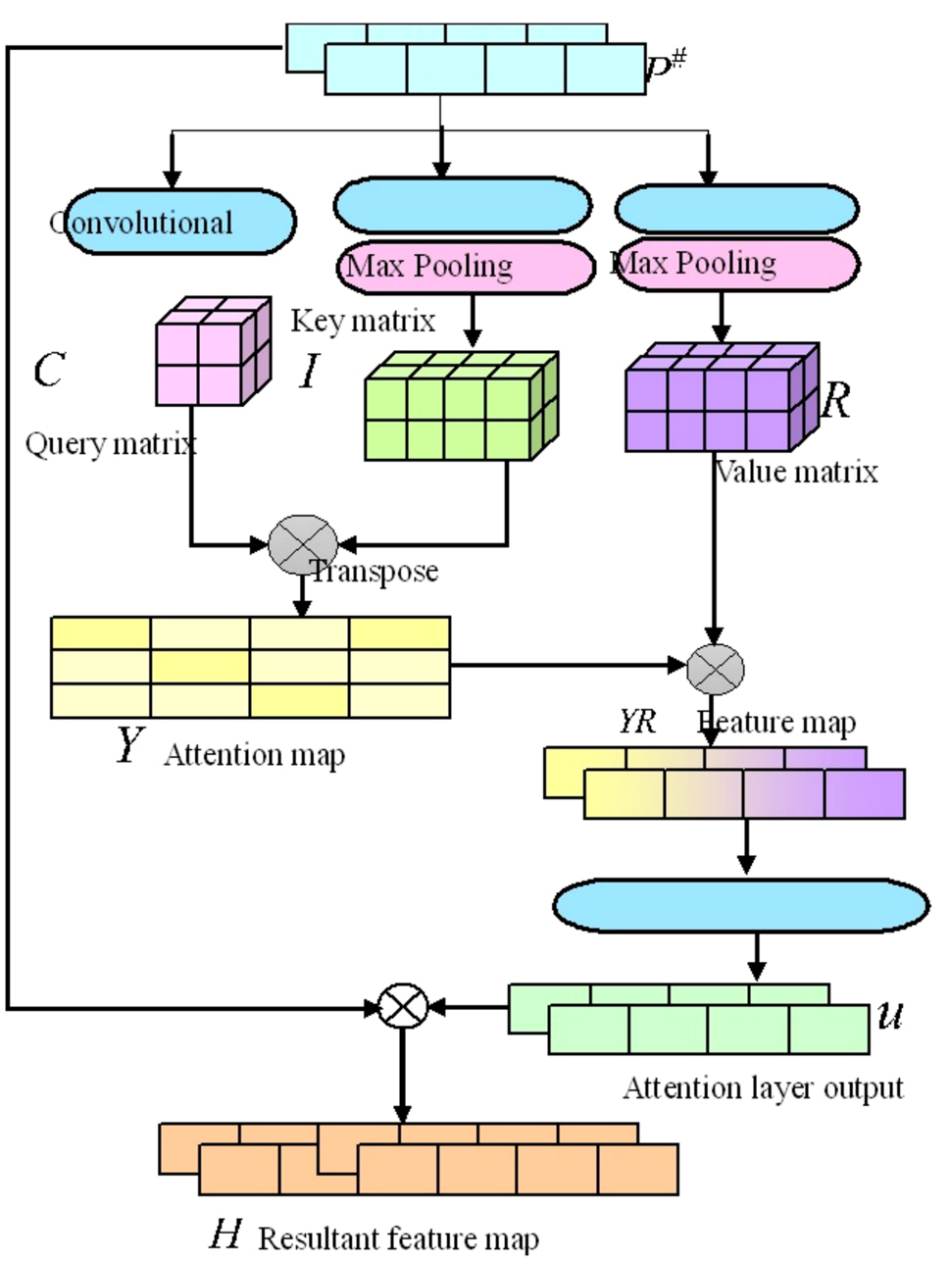
Architecture of the S$\vphantom{0}^{2}$A module.

In this research, the S$\vphantom{0}^{2}$A module is incorporated with the U-Net for the segmentation process, the ResNet-101 for the feature extraction process, and the RConvNet model for the BT classification process. The integration of the S$\vphantom{0}^{2}$A module helps to reduce the model’s number of parameters, and it facilitates the model to adaptively focus on the pixels present in the tumor region; therefore, the ability of feature representation of the model gets stronger, which results in an improved classification speed and accuracy. The internal working of the S$\vphantom{0}^{2}$A module is illustrated in Figure 4.

Primarily, the feature map $P^{\#}$, which is passed to the S$\vphantom{0}^{2}$A module, is transformed into a value matrix *R*, key matrix *I*, and query matrix *C* through the convolutional layer’s learned weight matrices. The number of values and keys is minimized by deploying a maxpooling layer to improve the efficiency of memory. The value, key, and query matrix are mathematically represented as,

15 $$\begin{aligned} R=(P^{\#})m^{R} \end{aligned}$$

16 $$\begin{aligned} I=(P^{\#})m^{I} \end{aligned}$$

17 $$\begin{aligned} C=(P^{\#})m^{C} \end{aligned}$$

where, $m^{R}$, $m^{I}$, and $m^{C}$ indicate the weight matrices of the convolution layer for the value, key and query matrix, respectively. After gaining the key, value, and query matrix, the attention map *Y* is obtained by fusing the query and the transpose of the key matrix through the SoftMax function, which is denoted as,

18 $$\begin{aligned} Y=SoftMax(CI^{t}) \end{aligned}$$

where $I^{t}$ denotes the transpose of the key matrix, and *Y* represents the attention map, which is then concatenated with the value matrix, *R* to attain the feature map $P^{\#}$. The obtained feature map$P^{\#}$ is passed into the layer of convolution to get the attention layer’s output *Y*, which is represented as,

19 $$\begin{aligned} U=(YR)m^{u} \end{aligned}$$

where $m^{u}$ indicates the weight matrix of the attention layer by convolution layer. Lastly, the attention layer’s output *Y* is fused with the original input, $P^{\#}$ with the learnable scalar parameter $\eta$ to gain the S$\vphantom{0}^{2}$A module’s resultant feature map, which is denoted as,

20 $$\begin{aligned} H=(\eta \times u)+P^{\#} \end{aligned}$$

where *H* signifies the resultant feature map from the S$\vphantom{0}^{2}$A module and $\eta$ denotes the learnable scalar parameter. Based on this same working procedure, the S$\vphantom{0}^{2}$A module performs in both the segmentation process and the feature extraction process. Pseudocode of the overall working Procedure of S2A-RConvNet model is explained in Algorithm 1.

**Table:** 

	**Pseudocode of the S2A-RConvNet model for BT classification**
Sl. No	Pseudo code for the S2A-RConvNet model
1.	Function: Brain Tumor classification
2.	Input: $S_{e}$ Patients’ MRI data
3.	Output: $q_{x}$ Predicted BT classification
4.	Begin
5.	Tumor Segmentation ()
6.	Return $D_{e}$ segmented tumor region.
7.	Process $Z_{e}$ ROI extraction
8.	Extract $\beta$ SRAG features
9.	Procedure proposed model (train data, test data)
10.	Model=sequential ()
11.	Model. Add (Conv2D)
12.	Model. Add (S2A module)
13.	Model. Add (Max_pooling2D)
14.	Compile (CCE loss= $X_{loss}=\sum ^{Z}_{x=1}g_{x}log(q_{x})$
15.	Return model
16.	Procedure testing model
17.	Out: model prediction (test data)
18.	Return Out $q_{x}$
19.	Return model

## Results and discussion

The dataset description, evaluation metrics, the S$\vphantom{0}^{2}$A-RConvNet model’s performance, and the obtained results are discussed in this part.

### Experimental setup

The S$\vphantom{0}^{2}$A-RConvNet model is implemented in a Python 3.7 tool on the Windows 11 OS, with an Intel Core i7 processor, 16GB of RAM, 128GB of ROM storage and 12GB GPU memory. The hyperparameters of S2A-RConvNet are demonstrated in Table 3.

**Table 3:** Hyperparameters.

Optimizer	Adam
Batch Size	32
convolutional layer filter size	16, 32, 64
Kernal size	3, 3
Max pooling: pool_size	2
strides	3
Dense	512, 128, 64
Loss Function	Categorial Cross-Entropy
Dropout rate	0.5
Learning Rate	0.001
Epochs	500
Activation Function	ReLU

### Dataset description

The BraTS 2021 dataset^25^ covers multi-parametric MRI scans, which are available as NIfTI files with 4 modalities, such as T2-weighted (T2), native (T1), T2-FLAIR, and post-contrast T1-weighted (T1Gd). There are 3 classes present with a total of 26754 samples of NCR, 7545 samples of ED, and 45745 samples of ET, respectively. In this research, 72040 images were used for training and 8004 for testing from the BraTS 2021 dataset.

**Fig. 5:**
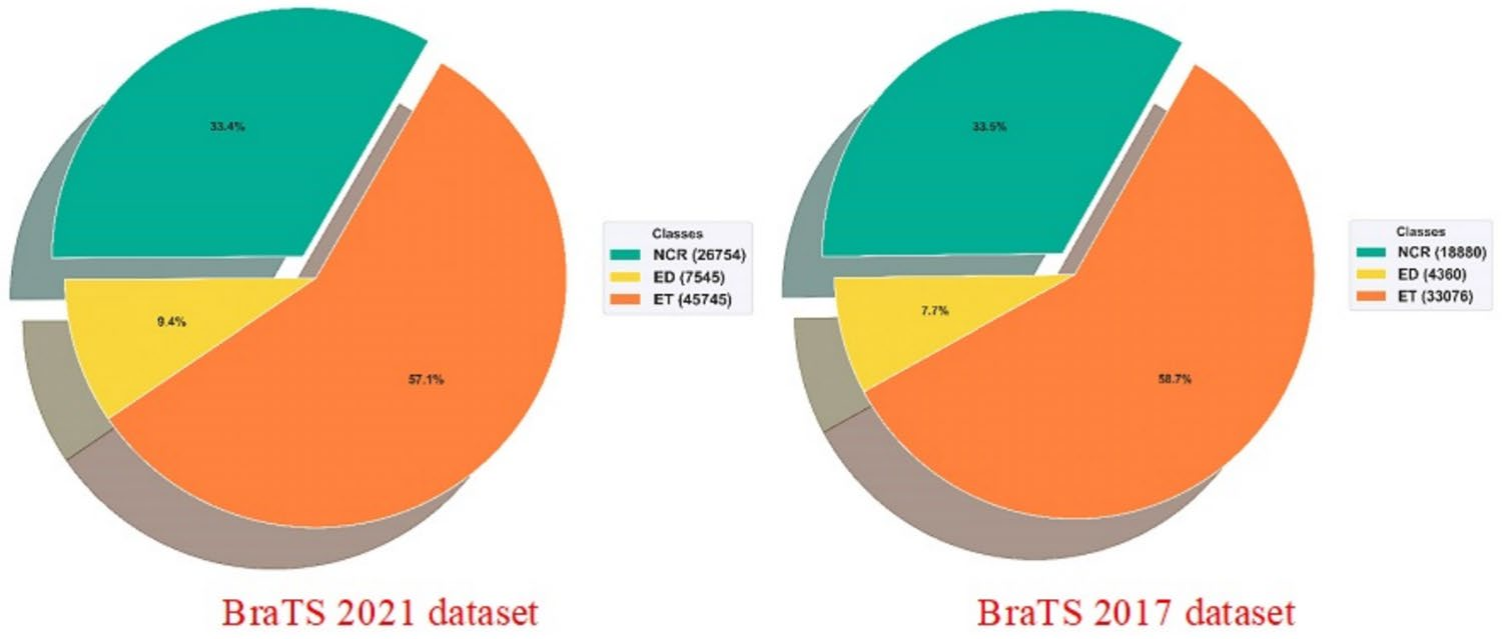
Dataset Visualization.

Similarly, the BraTS 2017 dataset^26^ includes a total of 56316 samples, and among them, 2460, 18880, and 33076 samples come under the classes of ED, NCR, and ET, respectively. From this dataset, a total of 50684 images were used for model training and 5632 images for testing purposes. Figure 5 shows the visualization of two datasets.

### Evaluation metrics

The S$\vphantom{0}^{2}$A-RConvNet model’s performance is estimated by the metrics, including precision, sensitivity, F1-score, specificity, and accuracy. The samples that are classified correctly are notated as $\psi$ and it is referred to true positives. The samples that are classified correctly as negative samples are denoted as $\lambda$ and it is named as true negatives. Similarly, the samples classified incorrectly are symbolized as $\chi$, which is termed as false positive, whereas samples incorrectly classified as negative samples are signified as $\mu$, and it is indicated as false negative, respectively. The mathematical formulation for the used metrics is as follows,

21 $$\begin{aligned} V_{1}=\frac{\psi +\lambda }{\psi + \lambda +\chi +\mu } \end{aligned}$$

22 $$\begin{aligned} V_{2}=\frac{\psi }{\psi + \chi } \end{aligned}$$

23 $$\begin{aligned} V_{3}=2 \times \frac{V_{2} \times V_{4}}{V_{2}+V_{4}} \end{aligned}$$

24 $$\begin{aligned} V_{4}=\frac{\psi }{\psi + \lambda } \end{aligned}$$

25 $$\begin{aligned} V_{5}=\frac{\lambda }{\lambda + \chi } \end{aligned}$$

where $V_{2}$ represents the precision, $V_{4}$ denotes the sensitivity, $V_{3}$ signifies the F1-score, $V_{5}$ indicates the specificity, and $V_{1}$ is the accuracy metric, respectively

### Image results

**Fig. 6:**
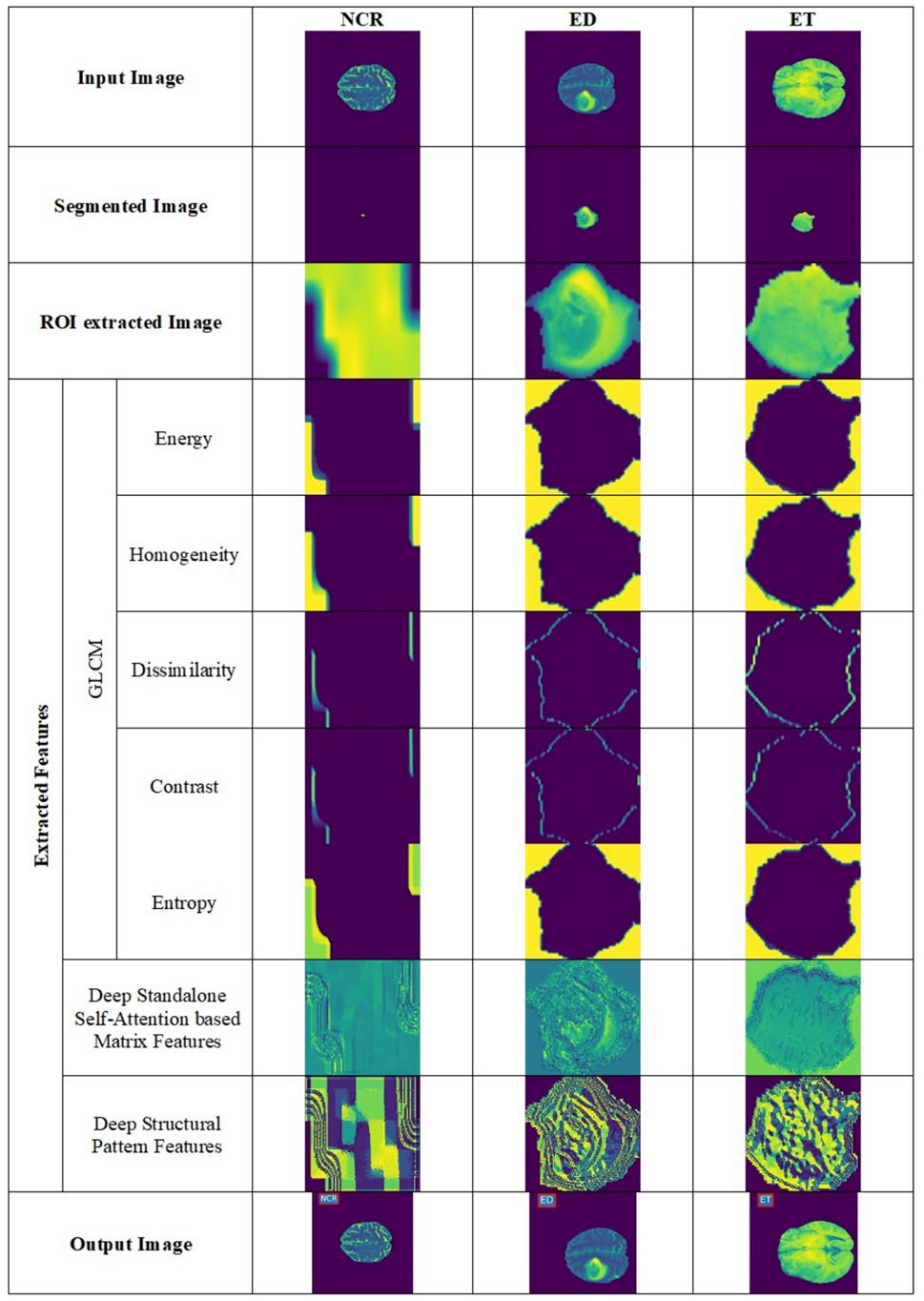
Image results using the BraTS 2021 dataset.

**Fig. 7:**
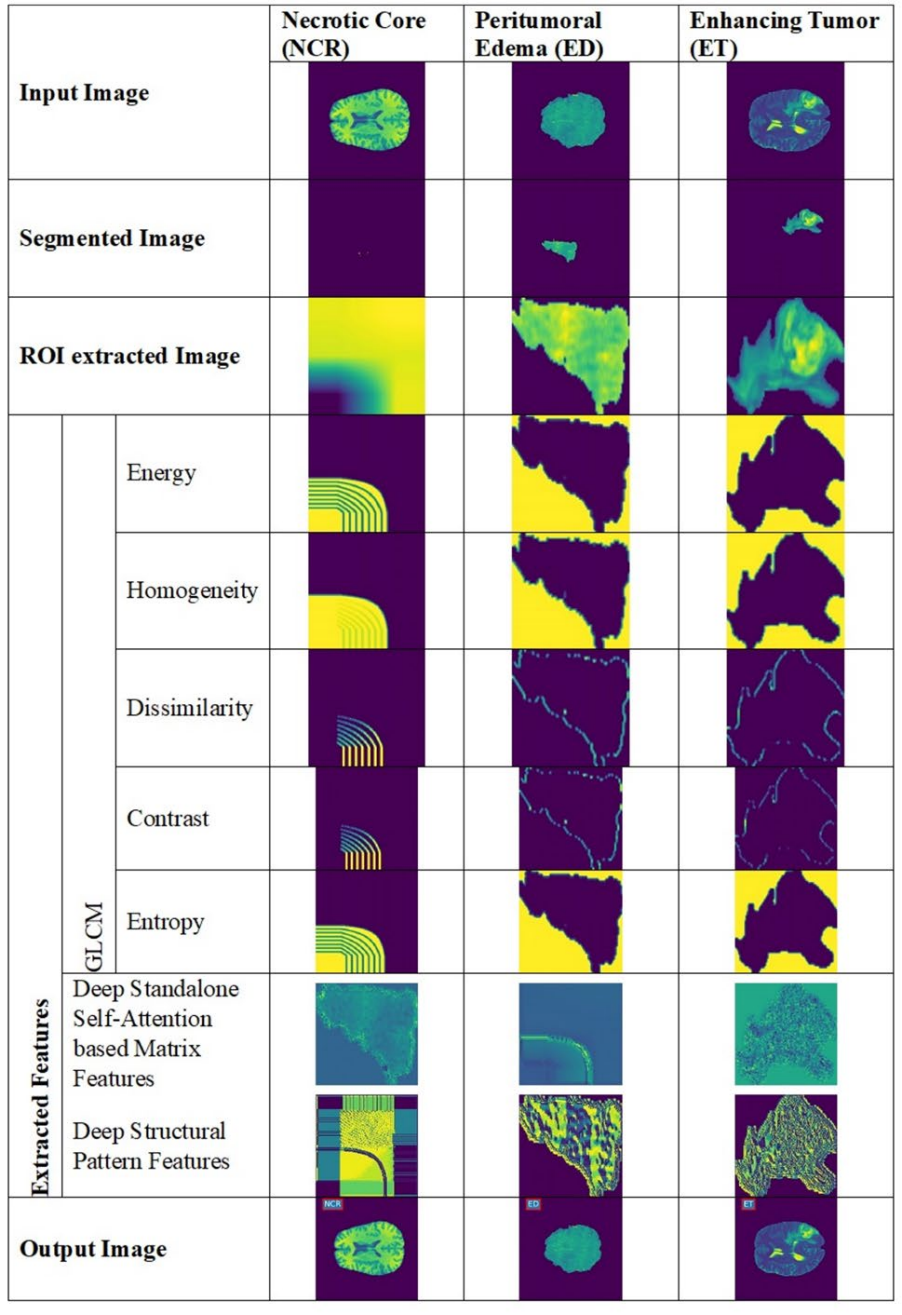
Image results using the BraTS 2017 dataset.

The sample results of the S$\vphantom{0}^{2}$A-RConvNet model obtained during every step in BT classification using the BraTS 2021 and BraTS 2017 datasets are revealed in Figures 6 and 7.

### Performance analysis

In this part, the S$\vphantom{0}^{2}$A-RConvNet model’s performance is analyzed by the BraTS 2021 as well as the BraTS 2017 datasets with multiple epochs, respectively.

#### Analysis of performance using BraTS 2021 dataset

**Fig. 8:**
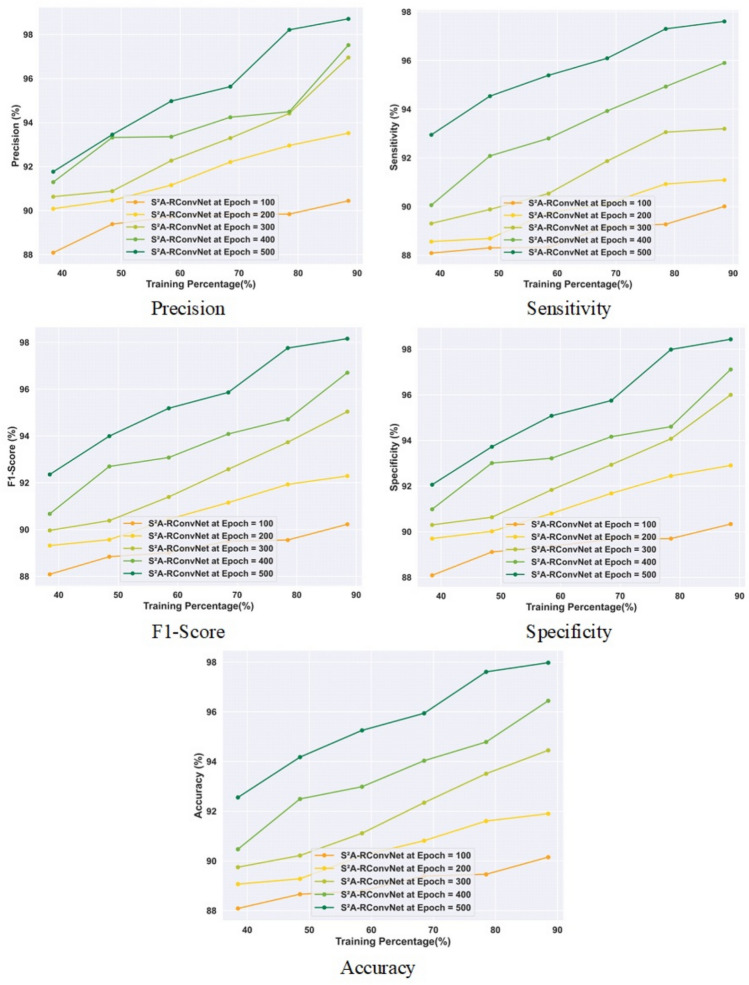
Performance of the S$\vphantom{0}^{2}$A-RConvNet model on the BraTS 2021 dataset.

Figure 8 reveals the performance of the S$\vphantom{0}^{2}$A-RConvNet in classifying BT using the BraTS 2021 dataset. The S$\vphantom{0}^{2}$A-RConvNet model gained a precision value at epochs 100 and 500 are 90.44% and 98.71%. The model’s sensitivity and F1-score at epoch 500 are 97.60% and 98.15%, respectively. The S$\vphantom{0}^{2}$A-RConvNet model’s specificity with a training set of 60% and 90% at epoch 500 is 95.07% and 98.43%. Similarly, the S$\vphantom{0}^{2}$A-RConvNet model’s accuracy value at epoch 200 is 91.89%, and at epoch 500 is 97.97%, respectively. The S$\vphantom{0}^{2}$A-RConvNet model attains better performance with increasing epochs and training data, and this result is obtained due to the incorporation of the S$\vphantom{0}^{2}$A module with the RConvNet that helps the model to capture the composite patterns and long-range dependencies, leading to improved and robust results in BT classification.

#### Analysis of performance using BraTS 2017 dataset

**Fig. 9:**
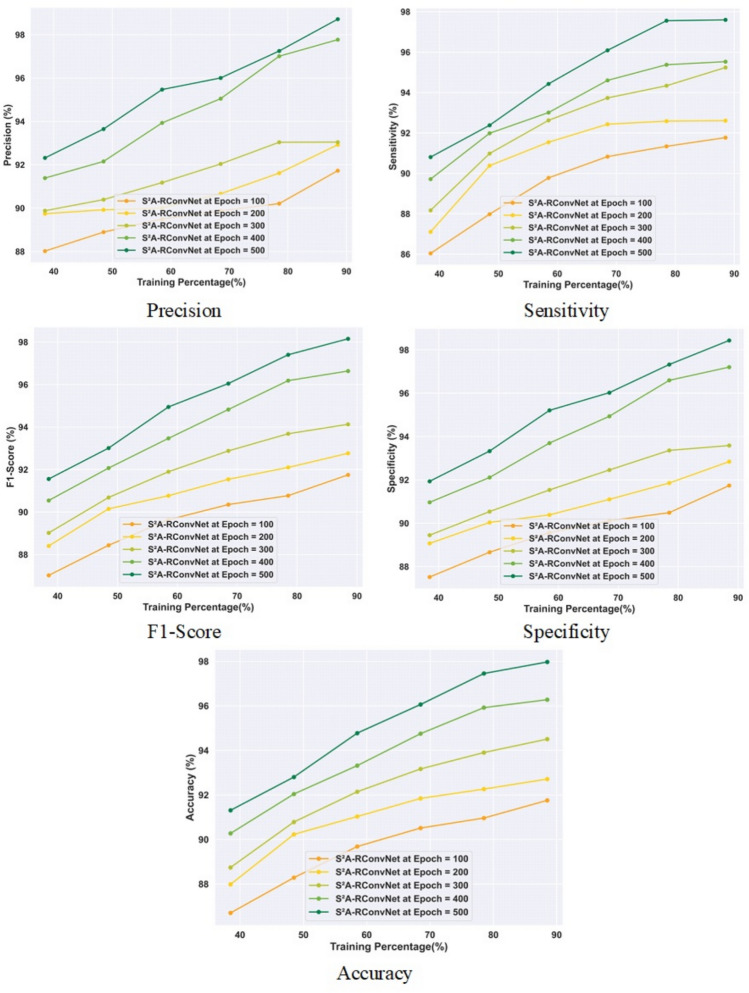
Performance of the S$\vphantom{0}^{2}$A-RConvNet model using the BraTS 2017 dataset.

The S$\vphantom{0}^{2}$A-RConvNet model’s performance in classifying BT by the BraTS 2017 dataset is shown in Figure 9. The precision value of 98.71% is attained through the S$\vphantom{0}^{2}$A-RConvNet model at epoch 500. The sensitivity of the S$\vphantom{0}^{2}$A-RConvNet model at epoch 500 with training data of 50% is 92.38% and training data of 90% is 97.69%, respectively. The F1-score of the S$\vphantom{0}^{2}$A-RConvNet model at epochs 500, 300, and 100 is 98.15%, 94.12% and 91.74% with 90% of training data. The S$\vphantom{0}^{2}$A-RConvNet model’s specificity is 91.73% with epoch 100, 92.84% with epoch 200, and 98.43% with epoch 500. Likewise, the accuracy of the S$\vphantom{0}^{2}$A-RConvNet at epochs 100 to 500 is 91.75%, 92.71%, 94.50%, 96.27% and 97.97%, respectively. The S$\vphantom{0}^{2}$A-RConvNet model attained better performance results on the BraTS 2017 dataset, which is due to the extraction of SRAG features, and it helps to diminish the computational complexity and boost the training process by extracting only the relevant features for BT classification.

### Comparative methods

The techniques used to compare with the S$\vphantom{0}^{2}$A-RConvNet model to evaluate its performance are EfficientNetB0^5^, EDN-SVM^2^, YOLOv5m^3^, Modified Inception V3^4^, Optimized CNN^6^, DCNN^7^, and ResNet-50^8^, based on various training percentage and K-fold values, which is explained in the following section.

#### Analysis of comparison using BraTS 2021 dataset with TP

**Fig. 10:**
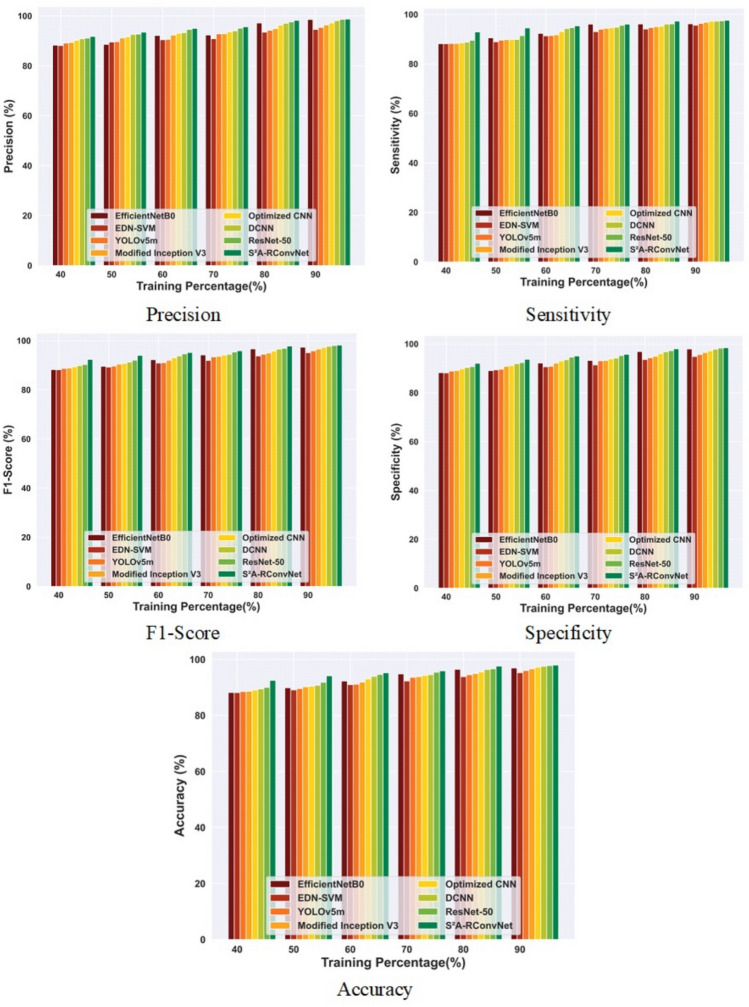
Comparison using the BraTS 2021 dataset based on TP.

The comparison of the S$\vphantom{0}^{2}$A-RConvNet model with other models using the BraTS 2021 Dataset for multiple training percentages is shown in Figure 10. At 90% training, the S$\vphantom{0}^{2}$A-RConvNet model attained high performance and gained a precision is 98.71%, which is 0.20% higher than the EfficientNetB0 model. The sensitivity of the S$\vphantom{0}^{2}$A-RConvNet is 97.61%, which is 1.46% greater than the EfficientNetB0 model. The F1-score and specificity of the S$\vphantom{0}^{2}$A-RConvNet model are 98.16% and 98.43%, which is 0.84% and 0.52% higher than those of the EfficientNetB0 model. Apparently, the proposed model reached a high accuracy of 97.98%, which is 1.04% higher than EfficientNetB0 and outperformed all the existing models. Therefore, the utilization of advanced models helped for accurate segmentation and BT classification, which improves the performance and reduces computational complexity.

#### Analysis of comparison using BraTS 2017 dataset with TP

**Fig. 11:**
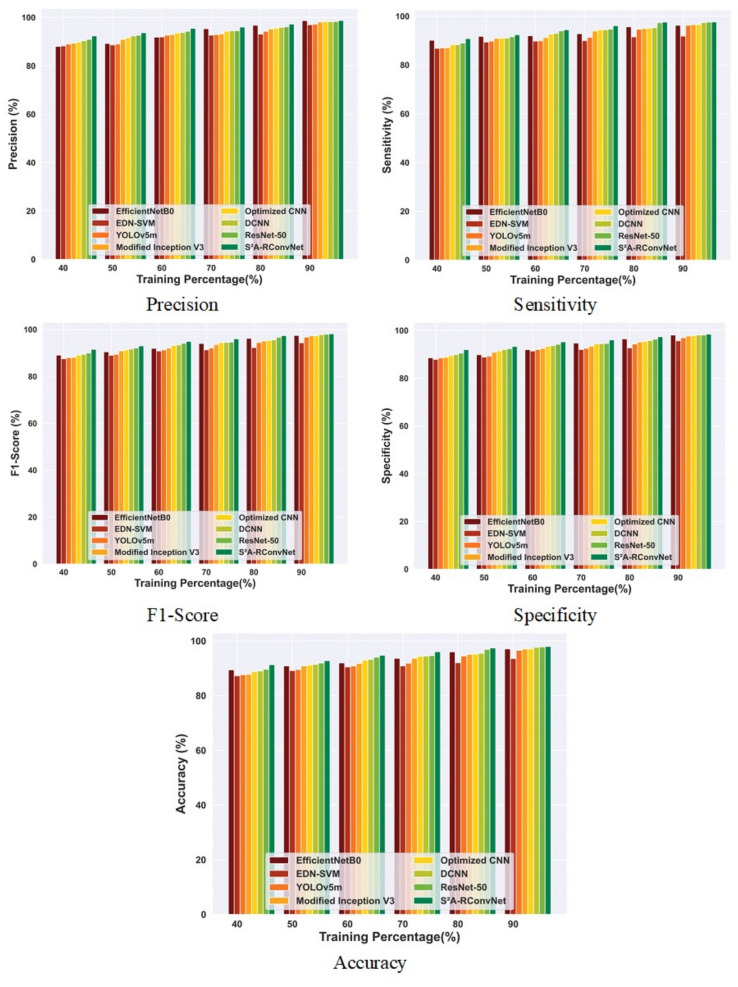
Comparative analysis using the BraTS 2017 dataset based on TP.

The S$\vphantom{0}^{2}$A-RConvNet model’s performance is evaluated against other models for multiple training percentages with the BraTS 2017 dataset is portrayed in Figure 11. The S$\vphantom{0}^{2}$A-RConvNet model gained superiority at 90% training and a precision is 98.72%, which is 1.79% greater than the EDN-SVM model. The sensitivity of the S$\vphantom{0}^{2}$A-RConvNet model is 97.60% and outperforms EDN-SVM with 5.92%. The F1-score of the S$\vphantom{0}^{2}$A-RConvNet model is 98.15% and the improvement percentage of 3.91% attained with EDN-SVM. The specificity and accuracy of the S$\vphantom{0}^{2}$A-RConvNet model are 98.44% and 97.97%, which is 2.84% and 4.53% improved over the EDN-SVM model. Similarly, the other existing methods are outperformed by the proposed model with its high performance in BT classification.

#### Analysis of comparison using the BraTS 2021 dataset with K-fold

**Fig. 12:**
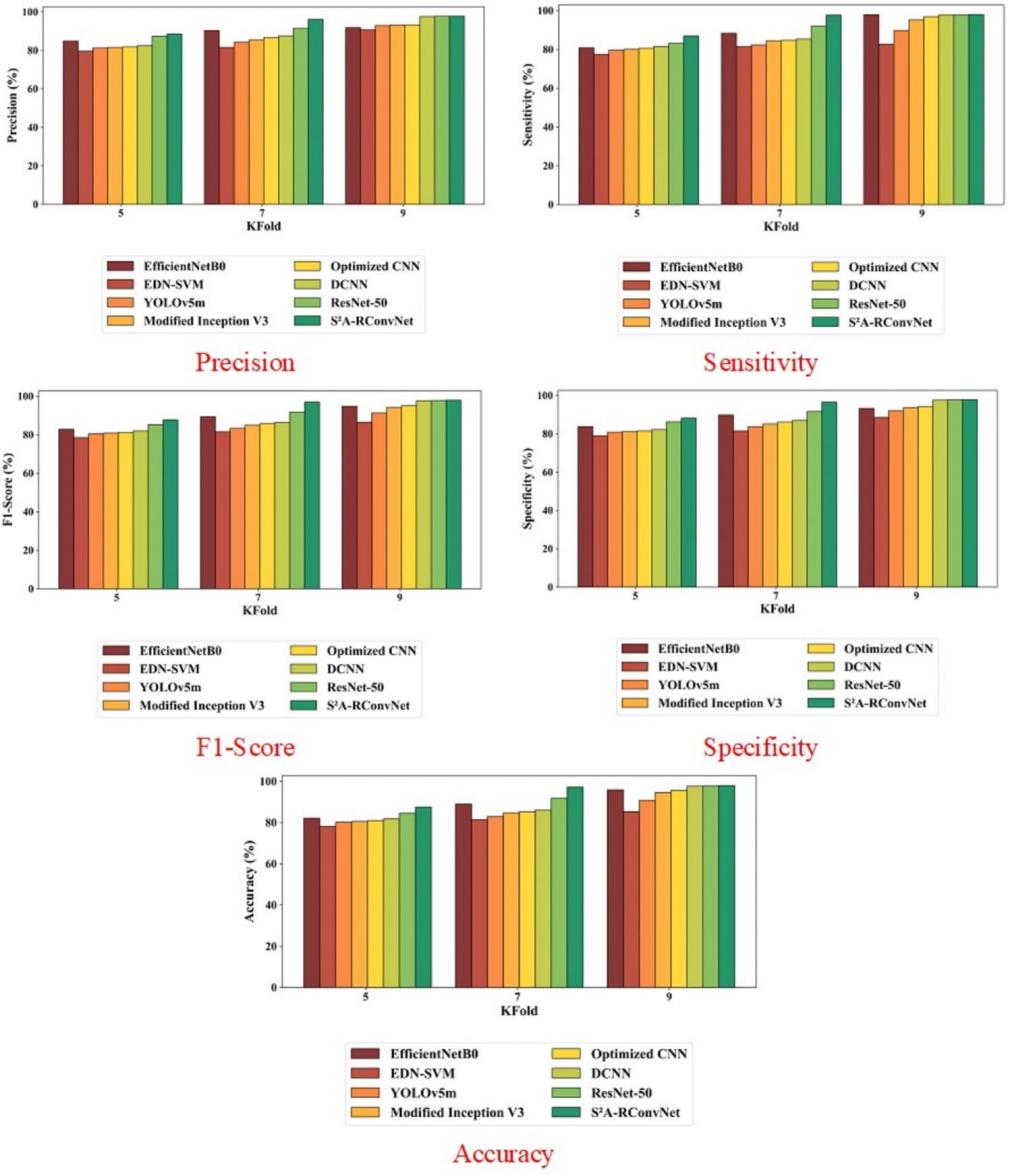
Comparative Analysis using the BraTS 2021 Dataset with K-fold.

The evaluation of the proposed S $\vphantom{0}^{2}$ A-RConvNet model is compared with other existing models based on multiple K-fold values using the BraTS 2021 Dataset, depicted in Figure 12. The proposed model achieved high performance at 9-fold values and attained a precision of 97.79%, which is 6.08% higher than the EfficientNetB0 model. The sensitivity of the S $\vphantom{0}^{2}$ A-RConvNet is 98%, which is 0.06% greater than the EfficientNetB0 model. The F1-score and specificity of the S $\vphantom{0}^{2}$ A-RConvNet model are 97.90% and 97.84%, which is 3.17% and 4.62% higher than those of the EfficientNetB0 model. Simultaneously, the proposed model reached a high accuracy of 97.93%, which is 2.06% higher than EfficientNetB0 and outperformed all the existing models. Therefore, the utilization of advanced models helped for accurate segmentation and BT classification, which improves the performance and reduces computational complexity.

#### Analysis of comparison using BraTS 2017 dataset with K-fold

**Fig. 13:**
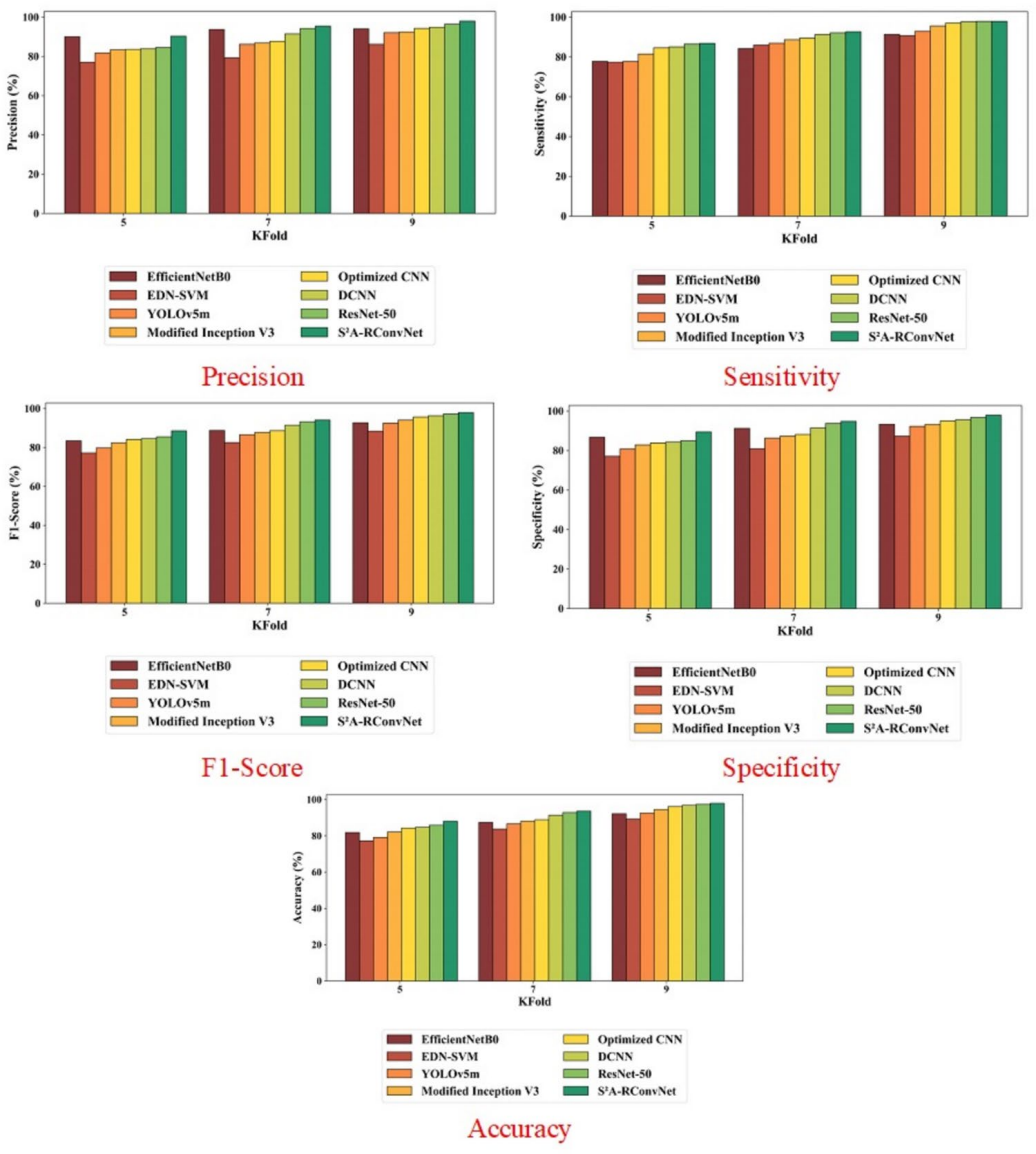
Comparative Analysis using the BraTS 2017 Dataset with K-fold.

The S $\vphantom{0}^{2}$ A-RConvNet model’s performance is evaluated against other models for multiple K-fold values with the BraTS 2017 dataset is depicted in Figure 13. The S $\vphantom{0}^{2}$ A-RConvNet model gained superiority at 9-fold and achieved a precision is 97.98%, which is 12.05% greater than the EDN-SVM model. The sensitivity of the S $\vphantom{0}^{2}$ A-RConvNet model is 97.93% and outperforms EDN-SVM with 7.28%. The F1-score of the S $\vphantom{0}^{2}$ A-RConvNet model is 97.96% and the improvement percentage of 9.73% attained over the EDN-SVM model. The specificity and accuracy of the S $\vphantom{0}^{2}$ A-RConvNet model are 97.97% and 97.95%, which is 10.89% and 8.88% improved over the EDN-SVM model. Similarly, the other existing methods are outperformed by the proposed model with its high performance in BT classification.

### Comparative discussion

The comparative methods EfficientNetB0, EDN-SVM, YOLOv5m, Modified Inception V3, Optimized CNN, DCNN and ResNet-50 provide numerous advantages in classifying BT, but due to the existence of some shortcomings, they are unable to achieve better results in classifying BT accurately. The performance drop in EfficientNetB0^5^ occurs due to the incapability of handling complex BT image features. The combination of deep neural and SVM in the EDN-SVM^2^ approach significantly increases the computational complexities, which leads to a longer training period. The YOLOv5m^3^ failed to classify the small tumor region; therefore, it is not suitable for an accurate BT classification. The Modified Inception V3^4^ model’s response time is higher, and it failed to adjust its knowledge for new circumstances. Due to the presence of overfitting issues in the Optimized CNN^6^, it failed to provide better results in classification. The DCNN^7^ model failed to extract the features with the existence of rich data; therefore, the model’s complexity is increased. The computational efficiency is poor in the ResNet-50^8^ model, respectively. The S$\vphantom{0}^{2}$A-RConvNet model overcame all the shortcomings of the comparative approaches, and due to the incorporation of the S$\vphantom{0}^{2}$A module, the proposed model can handle the complex BT image features. Moreover, extracting SRAG features helps to shorten the training period by extracting relevant features for classification. The higher time consumption issue in segmentation is solved by the S$\vphantom{0}^{2}$A-U-Net model for the segmentation process. The experimental results exhibited superior performance of the proposed S$\vphantom{0}^{2}$A-U-Net model in BT classification and surpassed other existing models with a high accuracy of 97.98%, Specificity of 98.43%, F1-Score of 98.16%, sensitivity of 97.61% and precision of 98.71% with the BraTS 2021 dataset at 90% training. Furthermore, the model’s performance is cross-validated with a K-fold value to exhibit consistent performance. The proposed model utilizes the strengths of the effective SRAG feature extraction, S$\vphantom{0}^{2}$A mechanism, segmentation-based S2A-U-Net, which attained less overfitting, fewer false errors and computational complexity. Overall, the S$\vphantom{0}^{2}$A-RConvNet model provides a more robust model for accurate BT classification and the comparative evaluation based on TP and K-fold using the BraTS 2021 and BraTS 2017 datasets is tabulated in Tables 4 and 5.

**Table 4:** Comparative Evaluation based on Training percentage.

Dataset		Methods/Metrics	EfficientNetB0^5^	EDN-SVM^2^ YOLOv5m^3^	Modified Inception V3^4^	Optimized CNN^6^	DCNN^7^	ResNet-50^8^	**S**$\vphantom{0}^{2}$**A-RConvNet**
BraTS 2021	Precision (%)	98.51	94.57	95.36	96.33	97.14	98.09	98.58	**98.71**
	Sensitivity (%)	96.18	95.63	96.35	96.80	97.27	97.30	97.41	**97.61**
	F1-Score (%)	97.33	95.10	95.85	96.57	97.21	97.69	97.99	**98.16**
	Specificity (%)	97.92	94.84	95.60	96.45	97.18	97.89	98.28	**98.43**
BraTS 2017	Precision (%)	98.65	96.95	97.19	98.08	98.20	98.25	98.30	**98.72**
	Sensitivity (%)	96.28	91.82	96.27	96.52	96.53	97.42	97.57	**97.60**
	F1-Score (%)	97.45	94.32	96.73	97.30	97.36	97.83	97.93	**98.15**
	Specificity (%)	98.05	95.64	96.96	97.69	97.78	98.04	98.11	**98.44**
	Accuracy (%)	97.07	93.53	96.57	97.04	97.09	97.69	97.81	**97.97**

**Table 5:** Comparative Evaluation based on Cross-validation.

Dataset		Methods/Metrics	EfficientNetB0^5^	EDN-SVM^2^ YOLOv5m^3^	Modified Inception V3^4^	Optimized CNN^6^	DCNN^7^	ResNet-50^8^	S2A-RConvNet
BraTS 2021	Precision (%)	91.85	90.71	92.95	93.19	93.31	97.61	97.77	97.79
	Sensitivity (%)	97.94	82.7	89.77	95.23	96.91	97.7	97.74	98
	F1-Score (%)	94.8	86.52	91.33	94.2	95.08	97.66	97.76	97.9
	Specificity (%)	93.32	88.61	92.14	93.69	94.19	97.63	97.76	97.84
	Accuracy (%)	95.91	85.37	90.83	94.55	95.71	97.67	97.75	97.93
BraTS 2017	Precision (%)	94.05	86.17	92.09	92.51	94.28	94.84	96.42	97.98
	Sensitivity (%)	91.33	90.79	92.92	95.62	97.07	97.79	97.92	97.93
	F1-Score (%)	92.67	88.42	92.5	94.04	95.66	96.29	97.16	97.96
	Specificity (%)	93.36	87.3	92.3	93.27	94.97	95.56	96.79	97.97
	Accuracy (%)	92.23	89.25	92.64	94.58	96.14	96.8	97.42	97.95

### Confusion matrix

**Fig. 14:**
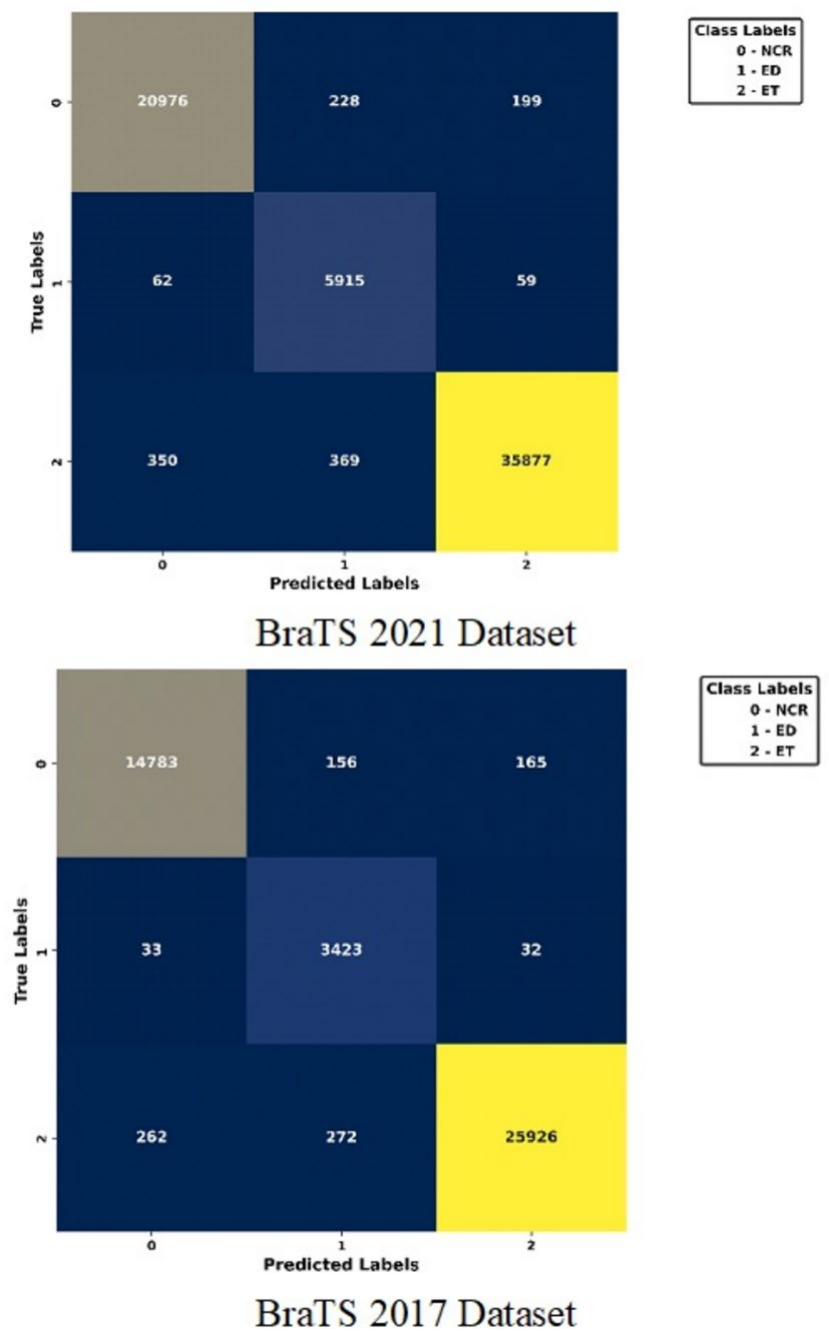
Confusion Matrix.

The S$\vphantom{0}^{2}$A-RConvNet model’s confusion matrix using BraTS 2021 and the BraTS 2017 datasets is illustrated in Figure 14. The confusion matrix analysis is performed to evaluate the S$\vphantom{0}^{2}$A-RConvNet model’s accurate prediction ability, and it summarizes the amount of correct and incorrect classification between true labels and predicted labels. According to this analysis, the model correctly predicted 20976 as NCR (0) classes, 5915 as ED (1) classes, and 35877 as ET (2) classes for the BraTS 2021 Dataset. Similarly, the model correctly predicted 14783 as NCR (0) classes, 3423 as ED (1) classes, and 25926 as ET (2) classes for the BraTS 2017 Dataset. In multi-class prediction, the model showed correctly predicted labels along the diagonal, while the remaining cells are considered as alternative classes rather than misclassifications, ensuring accurate performance evaluation and higher reliability

### ROC curve analysis

**Fig. 15:**
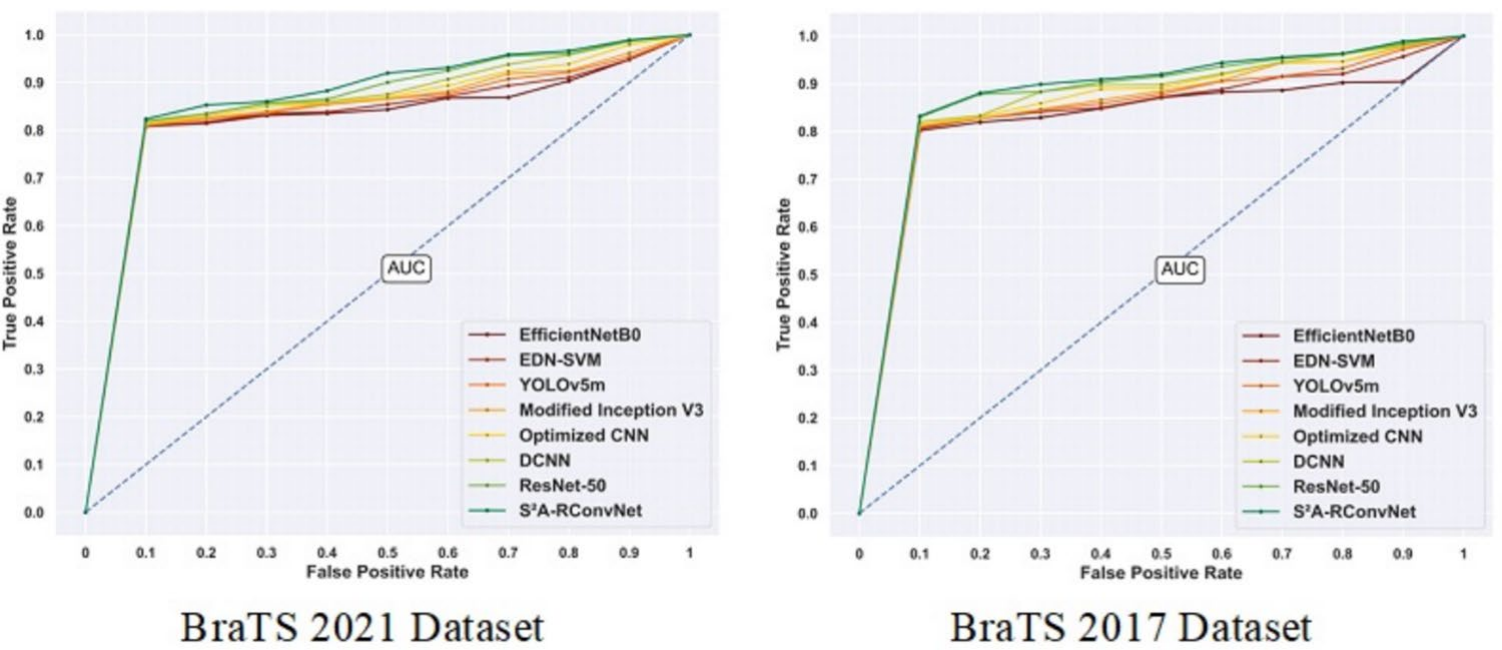
ROC Analysis.

Figure 15 represents the Receiver Operating Characteristic (ROC) curve of the S$\vphantom{0}^{2}$A-RConvNet and the comparative techniques using both BraTS 2021 and BraTS 2017 datasets. The ROC analysis demonstrates a trade-off between the True Positive Rate (TPR) and False Positive Rate (FPR) values within the range of 0 to 1. The effectiveness of the S$\vphantom{0}^{2}$A-RConvNet is depicted by the ROC plot, and the S$\vphantom{0}^{2}$A-RConvNet model attained an AUC value nearly equal to 1 and surpasses other methods, which signifies the outstanding proficiency of the model in classifying the BT accurately.

### PRC analysis

**Fig. 16:**
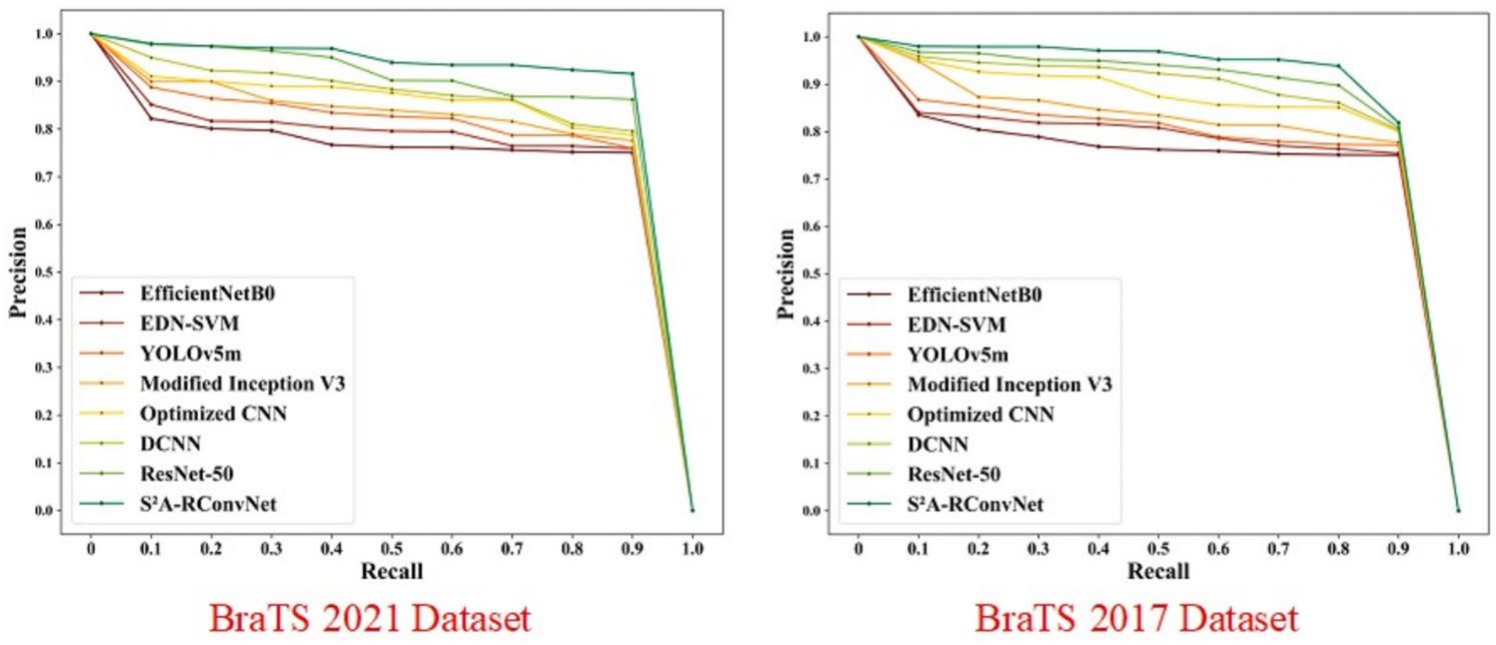
PR Curve Analysis.

Figure 16 explicates the precision-recall curve analysis for both datasets. The PRC analysis demonstrates a trade-off between the precision and recall values within the range of 0 to 1. When the PR curve is higher which indicates superior performance. Based on the graphical representations, the proposed S2A-RConvNet model proved high precision and recall values across multiple thresholds, demonstrating accurate BT classification. Moreover, the other existing models has less PR values, thereby attaining poor BT classification.

### Statistical analysis

The statistical test results attained by the S$\vphantom{0}^{2}$A-RConvNet model and the approaches are labelled in Tables 6 and 7. Furthermore, several statistical measures, including best, mean, and variance, are computed for the evaluation metrics of precision, F1-score, sensitivity, specificity and accuracy. The proposed S$\vphantom{0}^{2}$A-RConvNet model achieved a high best value in comparison to other existing models, demonstrating the significance of the model. Moreover, the statistical significance of the proposed model is evaluated with other existing models to detect the performance difference and significance of the model. Here, the proposed model is evaluated based on the T-statistic values and the P-value based on performance metrics using BraTS 2021 and BraTS 2017 Datasets. Specifically, the proposed model obtained p-values less than 0.05, which means the performance of the S$\vphantom{0}^{2}$A-RConvNet model is statistically significant.

**Table 6:** Statistical Test Results based on BraTS 2021 Dataset.

Methods/Metrics		Efficient NetB0	EDN-SVM	YOLO v5m	Modified Inception V3	Optimized CNN	DCNN	ResNet-50	S$\vphantom{0}^{2}$A-RConvNet
Precision	Variance	15.08	4.92	5.49	5.29	5.93	6.33	6.75	6.02
	Best	98.51	94.57	95.36	96.33	97.14	98.09	98.58	98.71
	Mean	92.81	91.15	91.93	92.79	93.64	94.30	94.92	95.46
	T-Statistic	2.62	3.06	2.73	3.34	3.15	3.10	3.31	3.37
	P-value	0.046	0.028	0.041	0.020	0.025	0.026	0.021	0.019
Sensitivity	Variance	9.81	7.16	8.20	8.97	9.28	9.76	7.64	2.56
	Best	96.18	95.63	96.35	96.80	97.27	97.30	97.41	97.61
	Mean	93.25	91.86	92.40	92.67	93.08	93.54	94.14	95.65
	T-Statistic	3.61	3.09	3.22	3.27	3.36	3.38	3.72	3.77
	P-value	0.015	0.027	0.023	0.022	0.020	0.019	0.0137	0.0130
F1-Score	Variance	11.45	5.85	6.72	6.90	7.36	7.62	7.03	4.09
	Best	97.33	95.10	95.85	96.57	97.21	97.69	97.99	98.16
	Mean	93.02	91.50	92.16	92.73	93.36	93.92	94.53	95.55
	T-Statistic	3.17	3.10	3.01	3.33	3.30	3.33	3.56	3.53
	P-value	0.024	0.026	0.029	0.020	0.021	0.0207	0.0161	0.0166
Specificity	Variance	13.03	5.34	6.08	6.04	6.57	6.86	6.85	5.00
	Best	97.92	94.84	95.60	96.45	97.18	97.89	98.28	98.43
	Mean	92.91	91.33	92.05	92.76	93.50	94.11	94.72	95.50
	T-Statistic	2.90	3.09	2.88	3.34	3.24	3.24	3.45	3.44
	P-value	0.033	0.027	0.034	0.020	0.0229	0.0228	0.0182	0.0183
Accuracy	Variance	10.70	6.25	7.19	7.54	7.93	8.21	7.19	3.54
	Best	96.96	95.27	96.02	96.64	97.23	97.56	97.80	97.98
	Mean	93.10	91.62	92.24	92.71	93.27	93.80	94.40	95.58
	T-Statistic	3.35	3.11	3.09	3.31	3.33	3.37	3.62	3.60
	P-value	0.020	0.026	0.027	0.021	0.020	0.019	0.0151	0.0154

**Table 7:** Statistical Test Results based on BraTS 2017 Dataset.

Methods/Metrics		EfficientNetB0	EDN-SVM	YOLOv5m	Modified Inception V3	Optimized CNN	DCNN	ResNet-50	S$\vphantom{0}^{2}$A-RConvNet
Precision	Variance	15.20	8.67	8.29	8.06	7.32	6.24	5.49	4.53
	Best	98.65	96.95	97.19	98.08	98.20	98.25	98.30	98.72
	Mean	93.29	91.90	92.50	93.26	93.82	94.19	94.45	95.57
	T-Statistic	3.03	2.81	2.73	3.11	3.35	3.41	3.33	3.41
	P-value	0.029	0.037	0.041	0.026	0.020	0.019	0.020	0.018
Sensitivity	Variance	4.76	2.68	9.70	9.74	7.46	8.66	9.20	6.51
	Best	96.28	91.82	96.27	96.52	96.53	97.42	97.57	97.60
	Mean	93.10	89.89	91.48	92.41	93.00	93.25	94.03	94.81
	T-Statistic	3.04	4.20	3.22	3.86	3.84	3.71	3.70	3.51
	P-value	0.028	0.008	0.023	0.011	0.012	0.0138	0.0139	0.017
Variance	8.99	4.82	8.62	8.64	7.30	7.37	7.10	5.41	0.35
	Best	97.45	94.32	96.73	97.30	97.36	97.83	97.93	98.15
	Mean	93.18	90.87	91.98	92.83	93.41	93.72	94.24	95.19
	T-Statistic	3.08	3.43	3.05	3.56	3.62	3.59	3.58	3.49
	P-value	0.027	0.018	0.028	0.016	0.0152	0.0157	0.0159	0.017
Specificity	Variance	11.85	6.54	8.35	8.28	7.28	6.78	6.23	4.94
	Best	98.05	95.64	96.96	97.69	97.78	98.04	98.11	98.44
	Mean	93.24	91.38	92.24	93.05	93.62	93.96	94.34	95.38
	T-Statistic	3.05	3.09	2.91	3.35	3.49	3.51	3.47	3.46
	P-value	0.028	0.027	0.033	0.020	0.0174	0.0171	0.0177	0.018
Accuracy	Variance	7.36	3.98	8.88	8.93	7.33	7.77	7.74	5.74
	Best	97.07	93.53	96.57	97.04	97.09	97.69	97.81	97.97
	Mean	93.16	90.56	91.82	92.69	93.27	93.56	94.17	95.06
	T-Statistic	3.08	3.67	3.12	3.67	3.70	3.63	3.63	3.50
	P-value	0.027	0.014	0.026	0.0143	0.0140	0.0150	0.0151	0.017

### Computational complexity

**Fig. 17:**
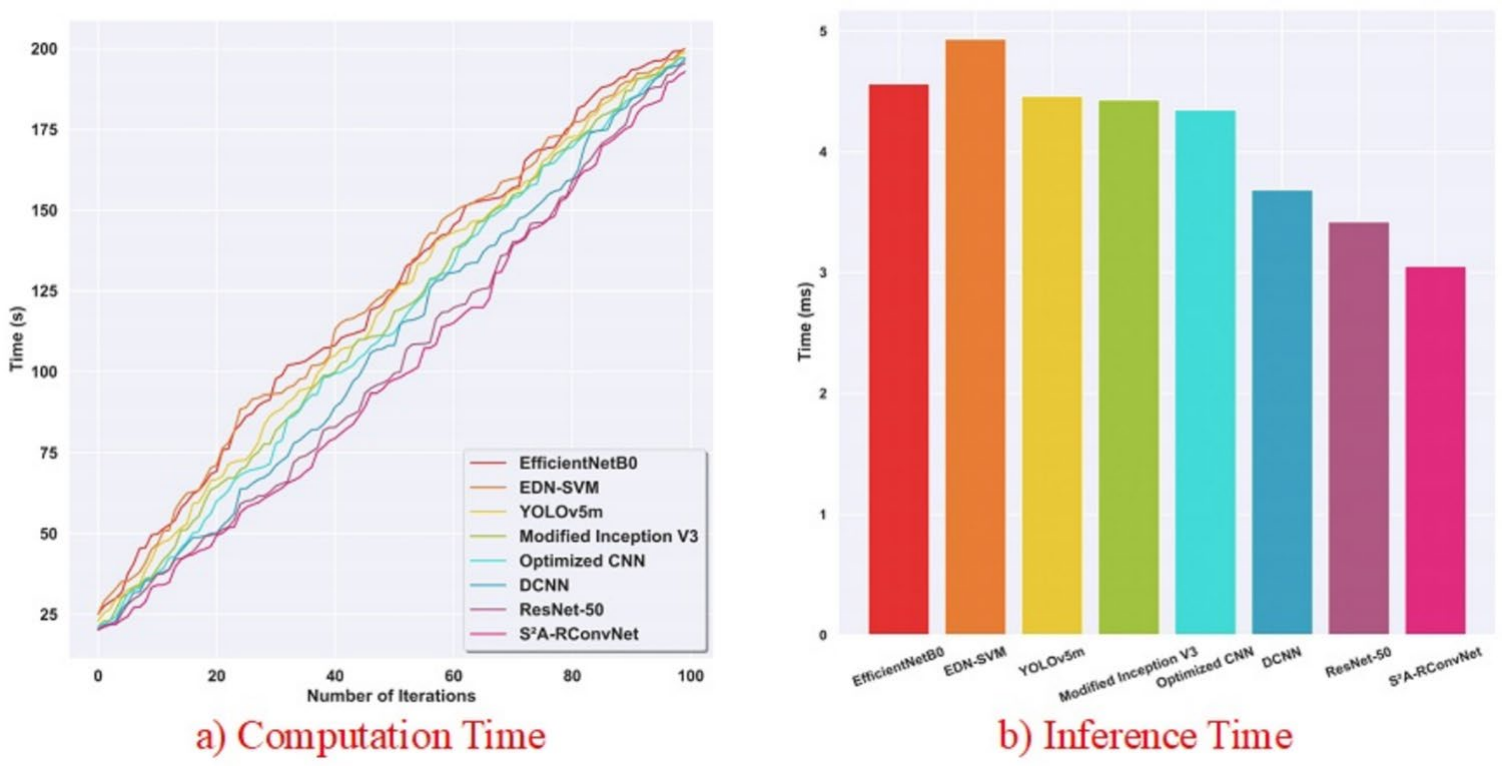
Computational Complexity Analysis.

Figure 17, a) demonstrates the computational time analysis of the S2A-RConvNet model compared with other existing models for multiple iterations. The required computation time of the proposed model for BT classification is 192.81s, which is less compared with other models. The other models utilized high computation time: EfficientNetB0 of 199.94s, EDN-SVM of 199.93s, YOLOv5m of 198.85s, Modified Inception V3 of 197.21s, Optimized CNN of 196.99s, DCNN of 196.57s, and ResNet-50 of 195.48s, respectively.

Figure 17 b) illustrates the inference time analysis of the S2A-RConvNet models compared with existing models. The proposed model achieved a faster inference time of 3.05ms and surpassed the EfficientNetB0 with 4.56ms, EDN-SVM with 4.93ms, YOLOv5m with 4.46ms, Modified Inception V3 with 4.43ms, Optimized CNN with 4.35ms, DCNN with 3.68ms, and ResNet-50 with 3.42ms, respectively. The utilization of the S2A module with the RConvNet model enhanced the classification accuracy and also learns complex features that reduce computational complexity.

### FLOPs analysis

**Fig. 18:**
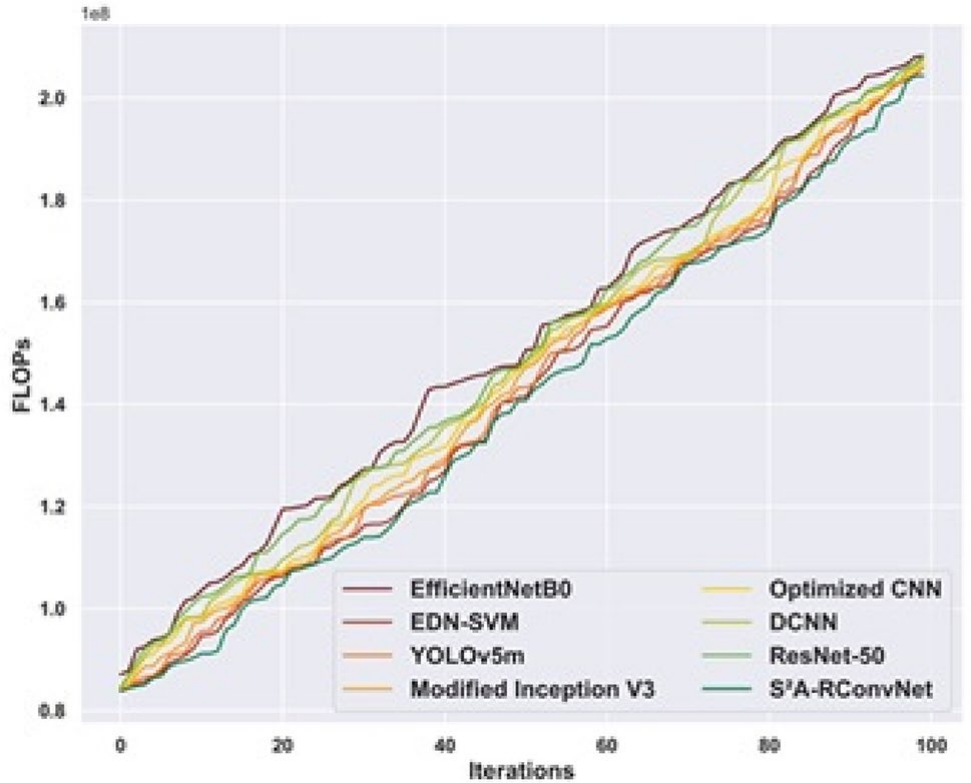
FLOPs Analysis.

FLOPs analysis explains the computational cost analysis in terms of Floating-point Operations Per second (FLOPs), which is depicted in Figure 18. Here, the analysis evaluates the model’s performance in handling the computational workload and resource utilization compared to other existing models. The proposed S2A-RConvNet model reduced the computation cost to $2.04\times 10^{8}$ FLOPs in 100 epochs. The other models attained high cost, such as EfficientNetB0 of $2.08\times 10^{8}$ FLOPs, EDN-SVM of $2.061\times 10^{8}$ FLOPs, YOLOv5m of $2.062\times 10^{8}$ FLOPs, Modified Inception V3 of $2.062\times 10^{8}$ FLOPs, Optimized CNN of $2.072\times 10^{8}$ FLOPs, DCNN of $2.076\times 10^{8}$ FLOPs, and ResNet-50 of $2.082\times 10^{8}$ FLOPs.

### IOU and DSC analysis for segmentation

**Fig. 19:**
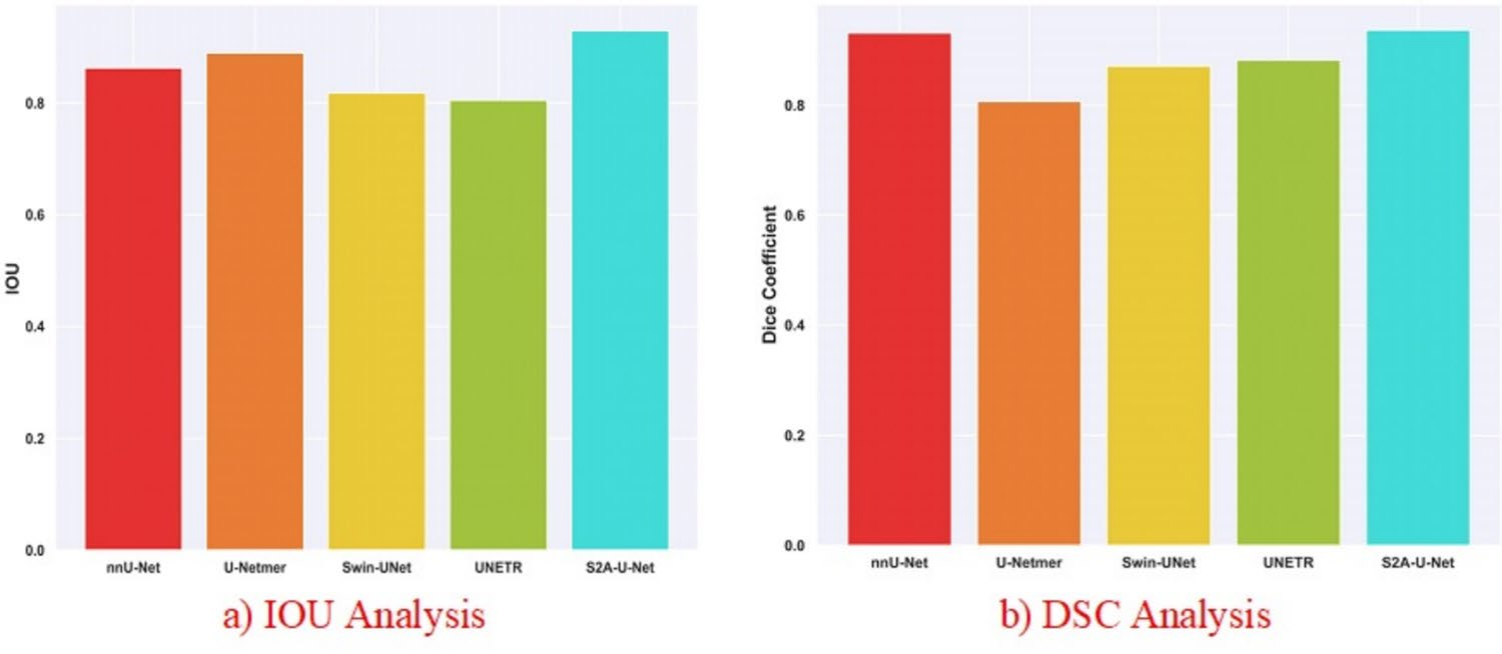
IOU and DSC Analysis for segmentation.

In this research, the segmentation is done by the S2A-U-Net model, which is compared with other existing segmentation methods to improve the detection accuracy, depicted in Figure 19, a. The Intersection over Union (IoU) metric evaluates the accuracy of BT segmentation by finding the ratio of the intersection area between the segmented region and the actual image under the union area. As per the analysis, the proposed S2A-U-Net model attained a high segmentation accuracy of 0.93 and outperformed other models, such as nnU-Net of 0.86, U-Netmer of 0.89, Swin-UNet of 0.82 and UNETR of 0.80.

The Dice Similarity Coefficient (DSC) evaluation for the S2A-U-Net model compared with other existing methods is depicted in Figure 19, b. The DSC is applied to analyze the segmentation performance by evaluating spatial overlap accuracy between the segmented region and the actual image. The DSC graph is plotted between 0 and 1, where 1 represents the better segmentation performance. As per the analysis, the proposed S2A-U-Net model attained a high segmentation accuracy of 0.94 and outperformed other models such as nnU-Net of 0.93, U-Netmer of 0.81, Swin-UNet of 0.87 and UNETR of 0.88. The integration of the S2A module with U-Net enhances the segmentation process while improving the model’s accurate BT classification.

### Evaluation loss curve analysis

**Fig. 20:**
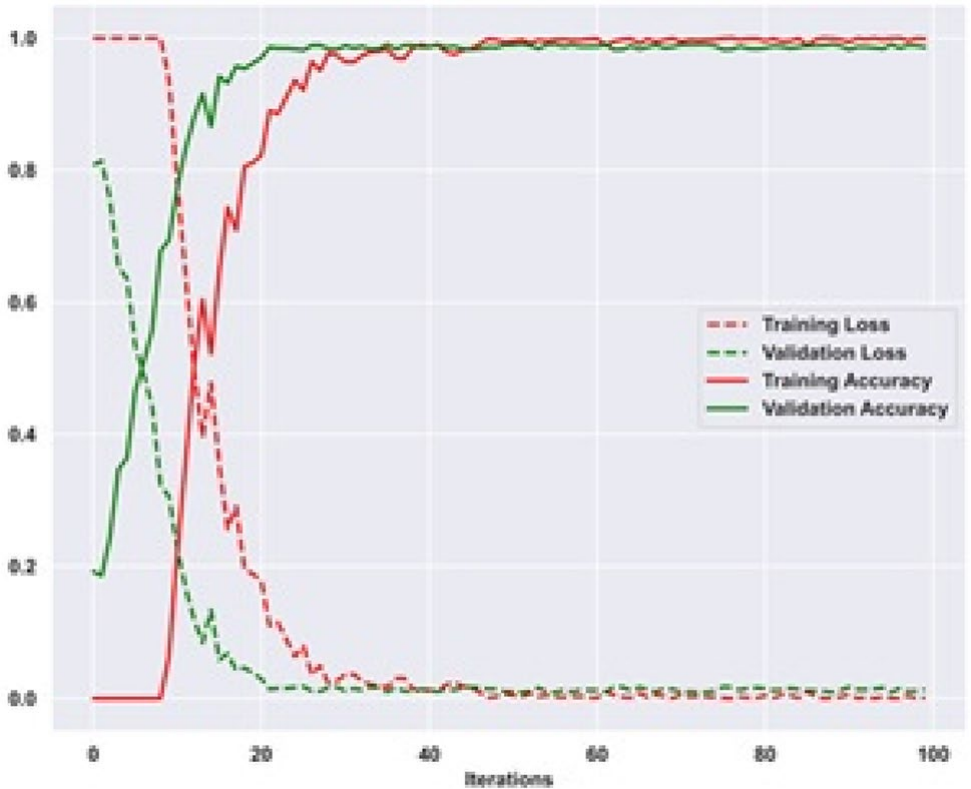
Evaluation Loss Curve Analysis.

The evaluation loss curves illustrating the S2A-RConvNet model’s training accuracy, validation accuracy, training and validation loss plotted for 100 iterations, depicted in Figure 20. When the epochs increase, both the training and validation accuracy of the model display a consistent growth of 0.986. Similarly, the training and validation loss of the model declined to 0.001 as the epochs increased. Moreover, the training and validation curves reveal the robust and accurate BT classification based on the minimum training loss and validation loss.

### Ablation study

**Fig. 21:**
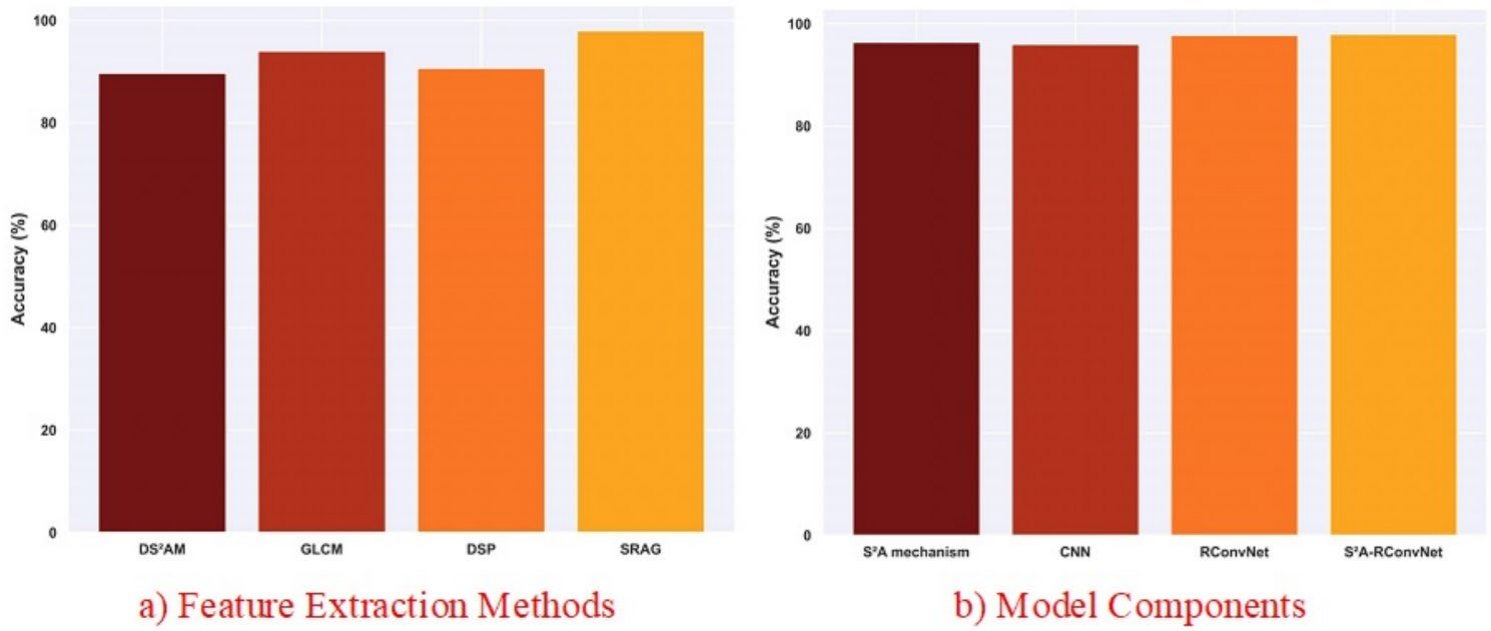
Ablation Study.

Figure 21 explicates the ablation study of the multiple feature extraction techniques and components used in the proposed S2A-RConvNet model for BT classification. The feature extraction methods, such as DSÂ$\vphantom{0}^{2}$AM, GLCM, DSP and SRAG, are compared based on accuracy, which is shown in Figure 21a. The methods obtained accuracy of 89.69%, 93.98%, 90.66%, and 97.98%. Here, the hybridising of all the feature extraction methods, SRAG obtained a high accuracy of 97.98% and outperformed other individual methods. Moreover, the application of these techniques extracts complex and important features that boost the accurate BT classification while reducing overfitting issues. Specifically, the depth feature extraction techniques and GLCM are used to differentiate the variations in photographic distortions and illumination changes from the MRIs. Therefore, these extracted features received from each technique are concatenated to generate significant SRAG features.

Figure 21, b) depicts the ablation study of multiple components used in the proposed S2A-RConvNet model based on accuracy. Based on the analysis, the S2A-RConvNet model attained a high accuracy of 97.98% and outperformed every component. The model components obtained less accuracy with the SÂ$\vphantom{0}^{2}$A mechanism of 96.37%, CNN of 95.96%, and RConvNet of 97.98%. Hence, the benefits of each component enhance the model’s performance, which leads to better robustness and scalability in BT classification.

## Conclusion

The S$\vphantom{0}^{2}$A-RConvNet model is proposed to solve the weaknesses of the conventional method in BT classification and to classify the BT accurately. The time-consuming drawback of the manual segmentation process is addressed by utilizing the S$\vphantom{0}^{2}$A-U-Net model for the segmentation process. Moreover, the incorporation of an ROI extraction mechanism decreased the computational load by exactly picking the tumor region, which also increases the training period. The extraction of SRAG features enhances the precision rate in the classification results of the S$\vphantom{0}^{2}$A-RConvNet model, improving the model’s performance and reducing computational complexity, which makes the model more robust than other conventional BT classification approaches. Furthermore, the repeated CNN can learn a hierarchy of complex features from the input, which leads to an accurate BT classification result. The values of precision, sensitivity, F1-Score, specificity, and accuracy achieved by the S$\vphantom{0}^{2}$A-RConvNet model are 98.71%, 97.61%, 98.16%, 98.43% and 97.98% with training data of 90% by the BraTS 2021 dataset, respectively. However, the absence of hybrid optimization hinders the model’s convergence and creates false errors. Hence, the advanced meta-heuristic optimization will be incorporated within the model in future research to optimize its hyperparameters to further improve its performance. Additionally, the explainable AI technique and advanced DL methods will be utilized to generate more accurate BT classification.

## Data Availability

The datasets analyzed during the current study are available in the BRaTS 2021 Task 1 Dataset, (https:/www.kaggle.com/datasets/dschettler8845/brats-2021-task1/data), and BraTS 2017 dataset, [https://www.kaggle.com/datasets/abdullahalmunem/brats17].
